# Evaluating Medical Entity Recognition in Health Care: Entity Model Quantitative Study

**DOI:** 10.2196/59782

**Published:** 2024-10-17

**Authors:** Shengyu Liu, Anran Wang, Xiaolei Xiu, Ming Zhong, Sizhu Wu

**Affiliations:** 1 Department of Medical Data Sharing Institute of Medical Information & Library Chinese Academy of Medical Sciences & Peking Union Medical College Beijing China

**Keywords:** natural language processing, NLP, model evaluation, macrofactors, medical named entity recognition models

## Abstract

**Background:**

Named entity recognition (NER) models are essential for extracting structured information from unstructured medical texts by identifying entities such as diseases, treatments, and conditions, enhancing clinical decision-making and research. Innovations in machine learning, particularly those involving Bidirectional Encoder Representations From Transformers (BERT)–based deep learning and large language models, have significantly advanced NER capabilities. However, their performance varies across medical datasets due to the complexity and diversity of medical terminology. Previous studies have often focused on overall performance, neglecting specific challenges in medical contexts and the impact of macrofactors like lexical composition on prediction accuracy. These gaps hinder the development of optimized NER models for medical applications.

**Objective:**

This study aims to meticulously evaluate the performance of various NER models in the context of medical text analysis, focusing on how complex medical terminology affects entity recognition accuracy. Additionally, we explored the influence of macrofactors on model performance, seeking to provide insights for refining NER models and enhancing their reliability for medical applications.

**Methods:**

This study comprehensively evaluated 7 NER models—hidden Markov models, conditional random fields, BERT for Biomedical Text Mining, Big Transformer Models for Efficient Long-Sequence Attention, Decoding-enhanced BERT with Disentangled Attention, Robustly Optimized BERT Pretraining Approach, and Gemma—across 3 medical datasets: Revised Joint Workshop on Natural Language Processing in Biomedicine and its Applications (JNLPBA), BioCreative V CDR, and Anatomical Entity Mention (AnatEM). The evaluation focused on prediction accuracy, resource use (eg, central processing unit and graphics processing unit use), and the impact of fine-tuning hyperparameters. The macrofactors affecting model performance were also screened using the multilevel factor elimination algorithm.

**Results:**

The fine-tuned BERT for Biomedical Text Mining, with balanced resource use, generally achieved the highest prediction accuracy across the Revised JNLPBA and AnatEM datasets, with microaverage (AVG_MICRO) scores of 0.932 and 0.8494, respectively, highlighting its superior proficiency in identifying medical entities. Gemma, fine-tuned using the low-rank adaptation technique, achieved the highest accuracy on the BioCreative V CDR dataset with an AVG_MICRO score of 0.9962 but exhibited variability across the other datasets (AVG_MICRO scores of 0.9088 on the Revised JNLPBA and 0.8029 on AnatEM), indicating a need for further optimization. In addition, our analysis revealed that 2 macrofactors, entity phrase length and the number of entity words in each entity phrase, significantly influenced model performance.

**Conclusions:**

This study highlights the essential role of NER models in medical informatics, emphasizing the imperative for model optimization via precise data targeting and fine-tuning. The insights from this study will notably improve clinical decision-making and facilitate the creation of more sophisticated and effective medical NER models.

## Introduction

### Background

The importance of robust named entity recognition (NER) models in medical informatics has become increasingly evident; these models systematically extract structured information from unstructured textual data such as clinical notes and research articles. This capability is crucial for processing large volumes of clinical data, facilitating early disease detection and supporting personalized medicine. NER models transform complex medical data into usable information, significantly enhancing clinical decision-making and medical research [1-3]. The performance of these models directly impacts the quality of information retrieval and data analysis as medical texts often contain complex and diverse terminologies. Precision in identifying and categorizing entities such as diseases, treatments, anatomical structures, and medications is foundational to many health care applications, making NER an indispensable tool in advancing medical informatics [4].

The critical role of NER in electronic health records further illustrates its importance. NER models automatically extract essential patient information, such as symptoms and medical conditions, that is crucial for differential diagnosis and ensuring that clinicians have rapid access to critical patient history. Errors in entity classification or omission can lead to severe consequences, including misdiagnosis or inappropriate treatment, highlighting the need for highly accurate NER models. Moreover, NER facilitates medical research by enabling efficient data mining from extensive medical literature, aiding the organization and retrieval of information on various medical entities. This capability is essential in drug discovery and identifying disease biomarkers, where systematic analysis and synthesis of large amounts of text are required. For instance, in pharmacovigilance, NER models identify adverse drug reactions from clinical notes and reports, contributing to drug safety monitoring and public health initiatives [5,6].

However, the application of NER in medical informatics presents unique challenges. The complexity and specificity of medical language, including synonyms, acronyms, and context-specific meanings, necessitate the continuous refinement of NER models. Recent studies have revealed significant variability in the effectiveness of NER models across different medical datasets, potentially limiting their real-world applicability. Despite these challenges, there have been promising advancements. For example, the BBC-Radical model, which integrates Bidirectional Encoder Representations From Transformers (BERT) with Bidirectional Long Short-term Memory, and conditional random field (CRF), has demonstrated high precision, recall, and *F*_1_-scores in extracting adverse drug reaction–related information from Chinese adverse drug event records [7]. This example illustrates the potential of combining advanced embedding techniques with traditional machine learning methods to enhance NER performance in specific contexts. These findings underscore the critical need for domain-specific adaptations and the integration of a more advanced linguistic and contextual understanding into these models. Such enhancements are essential for improving the prediction accuracy and applicability of NER models across diverse medical contexts.

Given these complexities and the need for continuous improvement, understanding the factors influencing model performance is crucial. Optimizing these models for specific tasks can significantly enhance their efficiency and accuracy. In medical informatics, this optimization is vital as the quality of data analysis directly impacts clinical decision-making. Researchers can make targeted improvements by identifying key elements of model design or aspects of training data that significantly affect performance, such as better handling of rare or complex medical terms. This knowledge assists in developing new models and refining existing ones to meet the specific needs of health care applications, thereby improving the precision of data extraction and analysis. Such improvements are crucial for the reliability of clinical information systems, reducing the risk of misdiagnosis or inappropriate treatment. Ultimately, these advancements support more accurate and informed clinical decision-making [8].

To achieve these improvements, it is essential to thoroughly evaluate the performance of various NER models across different contexts. First, this study identified 3 categories of NER models: statistical machine learning models, deep learning natural language processing (NLP) models based on BERT architecture, and large language models (LLMs). We selected these categories based on their unique strengths and potential to address specific challenges in medical NER.

Statistical machine learning models, such as hidden Markov models (HMMs) and CRF, were chosen for this study due to their established methodologies and effectiveness in sequence prediction tasks. These models leverage probabilistic approaches to capture the sequential nature of language data. However, they often struggle with the complexities of medical terminologies without extensive feature engineering. This limitation necessitates continuous refinement and adaptation to effectively handle medical texts’ intricate and specialized language [9].

Deep learning NLP models, especially those based on the BERT architecture, represent a significant advancement in NER capabilities. Variants of the BERT model, such as BioBERT, Robustly Optimized BERT Pretraining Approach (RoBERTa), Big Transformer Models for Efficient Long-Sequence Attention (BigBird), and Decoding-enhanced BERT with Disentangled Attention (DeBERTa), have demonstrated exceptional performance in capturing the intricate context of medical language. We selected these specific variants for this study because they can leverage deep contextual embeddings and undergo large-scale pretraining on medical corpora, enabling them to handle the complexities of medical terminologies effectively. For instance, a model using RoBERTa with whole-word masking and convolutional neural networks achieved high *F*_1_-scores in Chinese clinical NER tasks, indicating its effectiveness in processing complex medical terminologies within electronic medical records [10]. This success is primarily due to their ability to leverage deep contextual embeddings and undergo large-scale pretraining on medical corpora. These models are highly effective in identifying entities across diverse medical datasets, although they require substantial computational resources and meticulous fine-tuning for optimal performance. The introduction of BERT-based NER model variants marks a significant breakthrough in medical informatics, notably enhancing medical data analysis [11]. By leveraging advanced feature extraction techniques from masked language models such as embeddings from language models and the transformer architecture, these models set new standards for precise analysis of diverse medical datasets [12]. Specifically, RoBERTa incorporates enriched training data and refined masking patterns [13], whereas BigBird addresses previous models’ scalability and comprehension challenges by efficiently processing extended sequences [14]. DeBERTa’s innovative attention mechanisms further enhance these capabilities [15]. BioBERT, with its specialized pretraining on extensive medical texts, exemplifies the efficacy of domain-specific adaptations, achieving unparalleled precision in recognizing medical entities [16]. This strategic focus on contextual nuances and specialized terminology significantly improves the quality of patient care decisions.

LLMs, such as those based on the generative pretrained transformer architecture, have shown significant promise in NLP, particularly in understanding complex language structures. However, as the studies by Tian et al [17], Zhao et al [18], and Hu et al [19] have highlighted, their application in medical NER presents challenges, including limited prediction efficiency, extended runtimes, and substantial hardware requirements. These challenges are compounded by the complexity and specificity of medical terminology, often resulting in suboptimal accuracy with standard medical datasets such as BioCreative V CDR (BC5CDR), Joint Workshop on Natural Language Processing in Biomedicine and its Applications (JNLPBA), and NCBI (National Center for Biotechnology Information Disease Corpus). The general optimization strategies of LLMs do not align well with the specialized needs of NER tasks in medical informatics [17-19]. Despite these challenges, we selected Gemma, a fine-tuned version adapted explicitly for medical NER tasks, for this study [20]. Gemma was chosen over other open-source LLMs such as Large Language Model Meta AI 3 [21] or Open Pretrained Transformer [22] due to its advanced capabilities in contextual understanding and efficiency in processing specialized vocabulary. Its fine-tuning process specifically targets medical terminologies, making it more adept at handling the nuances of medical language. In addition, Gemma’s tailored adaptations include specific optimizations that align with the unique challenges of medical datasets, allowing it to achieve higher accuracy and reliability in recognizing entities within complex medical texts [23]. The proven effectiveness of fine-tuned models such as Gemma, as demonstrated by systems such as the Medical Named Entity Recognition–Japanese developed for analyzing pharmaceutical care records in Japanese, further underscores its suitability for medical NER tasks [24].

Building on this classification, we integrated various NER models, including HMM, CRF, RoBERTa, BigBird, DeBERTa, BioBERT, and Gemma. Following this integration, evaluating these models comprehensively to enhance their accuracy and durability is essential. Recent studies by Freund et al [25], Ahmad et al [26], and others highlight a shift toward more comprehensive evaluation metrics tailored to the specific needs of medical informatics. This shift facilitates the development of advanced NER applications, significantly improving patient care by enhancing clinical data management. This marks a substantial advancement in the integration of technology and health care. However, the evaluations’ primary focus has been assessing the predictive capabilities of NER models using metrics such as precision, recall, and *F*_1_-score. Research by Yoon et al [27], Yu et al [28], and Yadav and Bethard [29] has used these metrics to evaluate these capabilities. The study by Erdmann et al [30] also compared various NER tools for literary text corpora with human annotators using the same metrics, highlighting the importance of such evaluations in the digital humanities. Furthermore, the work by Usha et al [31] and Nagaraj et al [32], which includes advanced techniques such as confusion matrices and receiver operating characteristic and precision-recall curves, offers a more nuanced understanding of classification accuracy and errors in NER models. As the studies by Ozcelik and Toraman [33] and Akhtyamova [34] explored, error analysis is critical for understanding performance nuances, especially in identifying and categorizing short- and long-term entities. This comprehensive evaluation approach underscores the complexities and challenges in developing accurate and efficient NER models for medical informatics [33,34].

The aforementioned studies predominantly used standard metrics such as precision, recall, receiver operating characteristic curve, and *F*_1_-score to evaluate model performance. However, these metrics fall short in capturing performance variations across different medical NER datasets and conducting a detailed analysis of how dataset characteristics affect model performance. Moreover, although these metrics are easy to compute, their interpretation proves challenging. This difficulty mainly arises from the metrics’ failure to explain the broader reasons behind model outcomes as they often depend on processing microvector features that are not intuitively understandable. Consequently, researchers focused on enhancing medical NER datasets through NER models encounter significant hurdles in devising effective optimization strategies. In response, some researchers have shifted to customizing macrofactors for evaluating explanatory NER models. For example, Fu et al [35] developed an evaluation framework that outlines 8 distinct factor types to analyze their correlation with the models’ *F*_1_-score rankings. Zhou et al [36] proposed an ant colony optimization algorithm based on parameter adaptation. They designed a new dynamic parameter adjustment mechanism to adaptively adjust the pheromone importance factor. This algorithm is also suitable for selection of macrofactors. By adaptively changing the macrofactors, the algorithm can determine which macrofactors affect the prediction accuracy of the NER model [36]. Yao et al [37] also enhanced this domain with their groundbreaking a scale-adaptive mathematical morphology spectrum entropy algorithm, which adjusts the scale of structural elements to measure macrofactors’ impact on model prediction accuracy. These advancements have led to increasingly sophisticated NER model evaluations, resulting in more precise and resilient models.

### Objectives

Given this context, the purpose of this study was to systematically evaluate the comprehensive performance of various NER models in the medical field focusing on both general medical texts and specific medical entity types. In addition, this study aimed to explore key macrofactors affecting model prediction performance. To achieve these goals, we used a comprehensive evaluation approach combining traditional and innovative techniques to enhance the accuracy and reliability of NER in medical informatics. This approach included analyzing hardware performance indicators such as training duration, central processing unit (CPU), and graphics processing unit (GPU) use and assessing the precision of models such as HMM, CRF, RoBERTa, BigBird, DeBERTa, BioBERT, and Gemma across different medical entity types. Furthermore, we proposed the multilevel factor elimination (MFE) algorithm, which integrates linear and machine learning strategies to filter multilayer factors and evaluate their impact on prediction accuracy. Through this comprehensive evaluation, we aimed to provide targeted recommendations for researchers, ultimately leading to the development of more accurate and reliable NER models for broader applications in the medical field.

## Methods

### Overview

This section outlines 2 methods: *training and evaluating medical NER models* and *further assisted evaluation*. The first method evaluates the prediction accuracy of the statistical machine learning models (HMM and CRF), the deep learning NLP models based on BERT architecture (BioBERT, BigBird, DeBERTa, and RoBERTa), and the Gemma LLM across different medical entity types, as well as overall model effectiveness. The second method further assesses the prediction accuracy of merged entity types within these models’ postclassification and examines the macrofactors’ influence on model performance.

### Training and Evaluating Medical NER Models

#### Overview

This method involved training, validating, and testing NER models using hyperparameter tuning techniques. For models such as RoBERTa, BioBERT, BigBird, and DeBERTa, we used the Adaptive Moment Estimation (ADAM) optimizer for hyperparameter tuning. These strategies were applied using datasets such as the Revised JNLPBA, focusing on optimizing parameters such as learning rates, batch sizes, and other model-specific settings to enhance prediction accuracy. In addition, Gemma was fine-tuned using low-rank adaptation (LoRA) to improve its predictive performance. In contrast, models such as HMM and CRF were not subjected to hyperparameter tuning. This decision was based on the inherent simplicity of these models, which are less sensitive to hyperparameter variations than more complex deep learning models. Consequently, fine-tuning HMM and CRF would likely yield marginal improvements that did not justify the additional computational resources and time investment.

#### Dataset Selection

**Figure 1:**
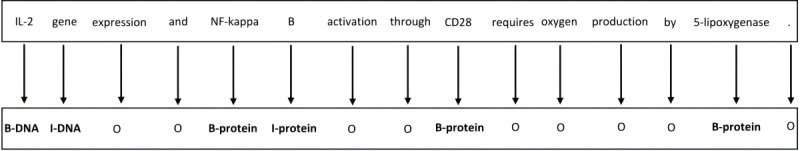
An instance of the beginning, inside, outside (BIO) sequence annotation method. CD28: cluster of differentiation 28; IL-2: interleukin-2; NF-kappa: nuclear factor kappa-light-chain-enhancer of activated B cells.

In total, 3 medical NER datasets were used to evaluate the prediction accuracy of models across different medical contexts (Table 1). These datasets use the “beginning, inside, outside” sequence annotation method (Figure 1); among them, the Revised JNLPBA dataset, provided by Huang et al [38], was selected because it retains the original semantic annotation type while addressing known vulnerabilities from the original JNLPBA dataset. It features 5 entity types (DNA, RNA, protein, cell line, and cell type), offering a focused scope on biological entities. The BC5CDR dataset, officially released by Li et al [39], was chosen for its 2 distinct entity types—disease and chemical—which present unique challenges due to their complex and overlapping terminology. Finally, the Anatomical Entity Mention (AnatEM) dataset [40], focusing on anatomical entities in medical fields, was used due to its broad range of 12 different entity types, providing a wide spectrum of medical terms. This diversity in entity numbers and types—most of which are distinct across the datasets—strengthens the evaluation by exposing the models to varied linguistic challenges, thereby reducing the randomness and contingency of the experimental results and enhancing the overall credibility of the experiment.

**Table 1:** Descriptive statistics of the medical named entity recognition datasets.

Dataset	Medical entity type	Entity types, n	Annotations, n
Revised JNLPBA^a^	DNA, RNA, protein, cell line, and cell type	5	52,785
BC5CDR^b^	Disease and chemical	2	38,596
AnatEM^c^	Organism subdivision, anatomical system, organ, multi-tissue structure, tissue, cell, developing anatomical structure, cellular component, organism substance, immaterial anatomical entity, pathological formation, and cancer	12	11,562

^a^JNLPBA: Joint Workshop on Natural Language Processing in Biomedicine and its Applications.

^b^BC5CDR: BioCreative V CDR.

^c^AnatEM: Anatomical Entity Mention.

#### Training and Testing of the Deep Learning NLP Models With BERT Architecture

##### Overview

This section discusses achieving optimal prediction accuracy through meticulous hyperparameter tuning during the model training phase. We trained models such as RoBERTa, BigBird, DeBERTa, and BioBERT on datasets divided into training, validation, and test sets sourced from the Revised JNLPBA, BC5CDR, and AnatEM collections. The ADAM optimizer was used to fine-tune key hyperparameters, including learning rate, batch size, epochs, and dropout rate. This optimizer adjusts the learning rate for each parameter based on estimates of lower-order moments of the gradients, enabling faster convergence by adapting to the characteristics of the data and gradients. This adaptive adjustment enhances convergence speed and overall model performance, aiming to identify the optimal hyperparameter combination that maximizes performance on the validation set [41,42].

The models’ prediction accuracy on the test set was evaluated primarily using *F*_1_-scores for specific entity types and overall performance metrics. The *F*_1_-scores, calculated as the harmonic mean of precision and recall, were chosen as the primary evaluation metric because they provide a balanced assessment of model performance in detecting relevant entities. This balance is crucial in medical NER, where precision measures the proportion of correctly identified entities and recall measures the proportion of actual entities correctly identified. In medical information extraction, where false positives and negatives can have significant implications, the *F*_1_-score’s ability to balance these metrics is essential. Missing important medical terms can lead to incomplete patient records or misunderstandings in medical literature, whereas false positives can introduce erroneous information into clinical decision-making processes. Thus, the *F*_1_-score is particularly suitable and reliable for evaluating model performance in medical NER tasks, where accuracy and reliability are paramount [43].

While the ADAM optimizer dynamically adjusted learning rates during model training to improve convergence, cross-validation was used separately to evaluate and optimize hyperparameters. Cross-validation, a robust method for model validation that involves dividing the dataset into multiple subsets for validation, ensures that the chosen hyperparameters generalize well to unseen data. This distinction underscores the complementary roles of the optimizer in training and cross-validation in validating and tuning the model’s hyperparameters. The selection and tuning of hyperparameters were guided by cross-validation. In this context, hyperparameter optimization aims to identify a global or satisfactory local optimum that maximizes medical NER model performance by adjusting several key hyperparameters, described in the following sections.

##### Learning Rate Adaptation Strategy

We used a dynamic learning rate adjustment strategy to enhance the model’s efficiency in converging to an optimal local minimum—testing rates of 0.0001 and 0.00001. This approach is similar to the findings of Wu and Liu [44] on the benefits of adaptive learning rates in NLP applications.

##### Batch Size Considerations

Guided by research on neural network training dynamics and computational constraints [44], we selected batch sizes of 10 and 50. This allowed for more frequent model updates and a finer approach to convergence.

##### Epoch Configuration

The number of training epochs was set based on the dataset’s complexity and initial performance metrics [45] with values of 1, 5, and 10. This adaptive approach minimized the risk of overfitting while ensuring the duration of practical training.

##### Dropout for Regularization

To prevent overfitting, we applied dropout rates of 0.1, 0.2, and 0.5 as informed by interim validation performance [46]. This regularization technique enhanced generalization across unseen medical texts, ensuring model reliability.

#### Training and Testing of the Gemma LLM

In this section, the Gemma 7B model was fine-tuned using the LoRA technique to optimize its performance on medical NER tasks. The LoRA approach was selected for its ability to efficiently adapt LLMs to specific tasks while reducing computational and memory overhead. This method involves freezing the pretrained model weights and introducing trainable low-rank decomposition matrices in each layer of the transformer architecture, significantly decreasing the number of trainable parameters and GPU memory requirements [47].

The fine-tuning process was conducted using a carefully structured setup. The Gemma 7B model was trained using datasets such as the Revised JNLPBA, BC5CDR, and AnatEM with a token cutoff length of 512 and a maximum of 20,000 samples. Preprocessing_num_workers was set to 16, and a mixture of depths was converted to facilitate diverse learning dynamics. The output configuration included saving checkpoints every 1000 steps and logging progress every 200 steps, ensuring thorough monitoring of the training process.

Training parameters included a per-device batch size of 8, a gradient accumulation step of 1, and a learning rate of 0.0001, managed by a cosine learning rate scheduler with a warm-up ratio of 0.1. The training spanned 3 epochs, using mixed precision with bfloat16 for efficiency. A weight decay of 0.01 was applied to prevent overfitting. A 10% validation split was used for evaluation, with assessments conducted every 200 steps using a batch size of 4 per device.

LoRA-specific settings included a dropout rate of 0.05 and a rank of 128 for the low-rank matrices applied across all model layers. This configuration enabled significant reductions in trainable parameters and GPU memory use. The LoRA technique allowed the Gemma 7B model to be fine-tuned effectively for complex NER tasks in the medical domain without compromising performance. These adjustments provided a scalable approach for adapting large-scale language models to specialized tasks with efficient computational resources.

Finally, the *F*_1_-score was used to evaluate the prediction accuracy of each entity type under the Gemma 7B model. To further demonstrate the effectiveness of the fine-tuning process, metrics such as the microaverage (AVG_MICRO) and macroaverage (AVG_MACRO) were leveraged to compare the performance of the fine-tuned model against a baseline without fine-tuning.

#### Training and Testing of the Statistical Machine Learning Models

This section used HMM and CRF for medical NER tasks using the Revised JNLPBA, BC5CDR, and AnatEM datasets. Unlike deep learning methods and LLMs, these statistical models did not require hyperparameter tuning. HMMs were trained by estimating transition and emission probabilities, whereas CRFs used feature functions to capture relationships between labels and features in the data. The prediction accuracy for each entity type was also evaluated using the *F*_1_-score.

#### Evaluative Metrics and Model Assessment

After the training, cross-validation, and testing phases, we used *F*_1_-score metrics across various hyperparameter combinations to evaluate the model’s prediction accuracy for different entity types. These *F*_1_-scores were aggregated into AVG_MICRO and AVG_MACRO as leveraged metrics to assess the model’s prediction accuracy under different hyperparameter configurations. The AVG_MICRO metric provides a comprehensive measure of overall model performance, capturing precision and recall across the dataset. The AVG_MACRO metric highlights the model’s ability to handle varying entity distributions, including rare entities. This dual-metric approach ensures a balanced evaluation, preventing biases toward more common entities and maintaining consistent performance across diverse entity types [42].

To enhance our evaluation framework, we assessed training time efficiency and computational resource use, including CPU and GPU use. These additional metrics provide insights into each model’s operational demands and feasibility, allowing us to evaluate their prediction accuracy and practicality for real-world applications.

### Further Assisted Evaluation

#### Overview

This method further evaluated the relationship between medical data and NER models, focusing on categorical relaxation to reduce entity classification complexity and improve prediction accuracy. We detailed the process of merging similar entity types into broader categories and evaluated the impact on model performance through a comprehensive analysis of macrofactors such as sentence length (sLen) and entity density (eDen). These methods were applied across several datasets, such as Revised JNLPBA, BC5CDR, and AnatEM, to systematically assess NER models.

#### Academic Revision on Categorical Relaxation

Categorical relaxation in NER classification, particularly within the medical domain, entails merging similar medical entity types to diminish ambiguity and enhance the classification performance of NER models. This technique simplifies the classification landscape and bolsters the models’ capacity to generalize from training data to unseen clinical examples. In this study, we implemented categorical relaxation by consolidating specific medical entity types, such as merging different DNA or RNA names. This method was guided by empirical evidence suggesting that merging reduces misclassifications and boosts prediction accuracy, particularly in diagnosing conditions and recommending treatments.

In the Revised JNLPBA dataset, we adopted a merging strategy based on the principles described by Tsai et al [48]. Biologically related categories such as DNA, RNA, and proteins were consolidated into a single “macromolecule” category. Similarly, entities categorized as cell lines and cell types were combined into a single “cell” entity category.

In the AnatEM dataset, we reclassified 12 entity types into 4 broader categories relevant to human health following the classification guidelines from Pyysalo and Ananiadou [40]. This reorganization is predicated on the premise that broader categories more effectively capture essential information and reduce the noise caused by particular and infrequently occurring entities.

Following categorical relaxation, we comprehensively evaluated the RoBERTa, BigBird, DeBERTa, and BioBERT models. This assessment compared the prediction accuracy of these models on the newly consolidated entity types. The objective was to determine the effects of entity type consolidation on model performance, particularly whether simplifying categories enhanced prediction accuracy across different model architectures. This method simplifies entity classification and capitalizes on the inherent similarities among entity types to improve model training and evaluation. By reducing the granularity of entity types, we posited that the models would achieve higher accuracy and more effectively address the complexities of medical texts. The specific outcomes of this categorical relaxation are detailed in Table 2.

**Table 2:** Merged entity types.

Dataset and medical entity type			Merged entity type
**Revised JNLPBA^a^**			
	DNA, RNA, and protein	Macromolecule	
	Cell line and cell type	Cell	
**BC5CDR^b^**			
	Disease	Disease	
	Chemical	Chemical	
**AnatEM^c^**			
	Organism subdivision, anatomical system, organ, multi-tissue structure, tissue, cell, developing anatomical structure, and cellular component	Anatomical structure	
	Organism substance	Organism substance	
	Immaterial anatomical entity	Immaterial anatomical entity	
	Pathological formation and cancer	Pathological formation	

^a^JNLPBA: Joint Workshop on Natural Language Processing in Biomedicine and its Applications.

^b^BC5CDR: BioCreative V CDR.

^c^AnatEM: Anatomical Entity Mention.

#### Constructing and Evaluating NER Macrofactor Datasets

##### Macrofactor Metrics Definition

Entity macrofactor metrics were defined in an entity (entity phrase fragment) or on the entire dataset, and dataset’s different attributes were described. On the basis of the 8 factor types defined by Fu et al [35], we categorized these into 6 macrofactor metrics. This categorization was derived from a detailed examination of the characteristics of 3 medical datasets, including the length of medical terminology and other relevant factors. This allowed us to distill the most pertinent metrics for our study, which included *sLen*, entity phrase length *(eLen)*, *eDen*, number of entity words in each entity phrase (*eNum*), total entity word count in each entity type *(tEWC)*, and entity label consistency *(elCon)* (Textbox 1)

**Definitions of macrofactor metrics.**

- *sLen*: this metric refers to the string length of the sentence containing the entity phrase. It quantifies the contextual space in which entities appear. Notably, extreme values are excluded to prevent distortion in average calculations.
- *eLen*: this metric quantifies the string length of each entity phrase, which can comprise one or more entity words. Similar to *sLen*, extreme values are excluded to yield a more precise average *eLen*for each entity type.
- *eDen*: this metric is calculated as the ratio of *eLen* to *sLen*; this metric quantifies how dense entities are populated within the text.
- *eNum*: for datasets such as Revised JNLPBA labeled with the “beginning, inside, outside” sequence, this metric counts the entity words in a phrase, adjusting for labeling specifics such as combining “B-Entity label” and “I-Entity label” into a single count to avoid underrepresentation in the data.
- *tEWC*: this metric quantifies the cumulative number of entity words within each specific entity type across datasets such as the Revised JNLPBA, BC5CDR, and AnatEM.
- *elCon*: this metric evaluates the consistency of entity-type assignments to medical terms across various contexts, which is essential in datasets such as the Revised JNLPBA dataset, where terms can possess multiple semantic interpretations. For instance, “lymphocyte” may be categorized as “B-cell_type” in discussions about receptor counts and as “I-cell_type” in contexts involving blood samples. To calculate*elCon*, each term from the dataset is cataloged along with its associated entity types. For example, “lymphocyte” would be documented with both “B-cell_type” and “I-cell_type.” A weight ω is assigned to each term based on the inverse number of its entity types (ω=1/number of entity types), indicating that terms linked to fewer entity types tend to demonstrate higher prediction accuracy. Terms associated with a single entity type are assigned a weight of 1, whereas those with 2 types are given a weight of 0.5. We calculate the average weight using the following formula—average weight value 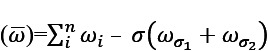
—excluding extreme values to avoid data skew.

##### The New Macrofactor Dataset Creation

**Figure 2:**
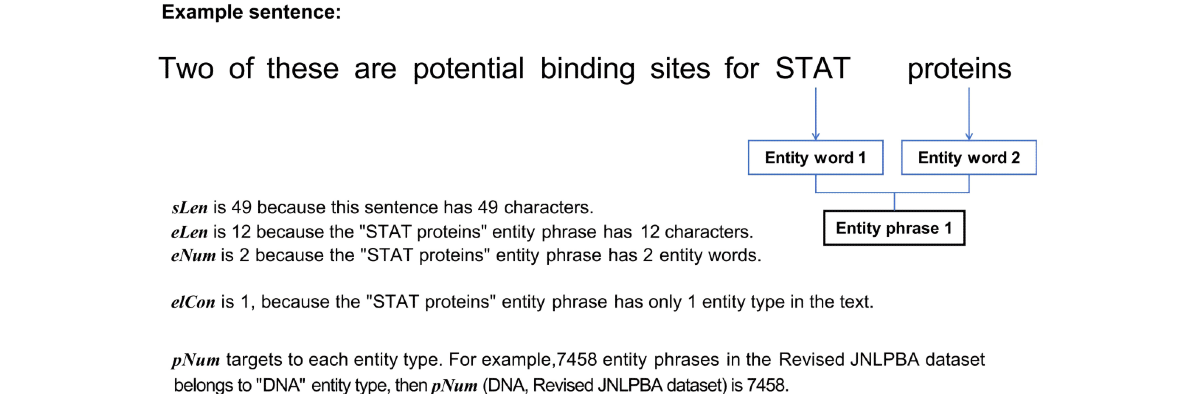
Sentence length (sLen), entity phrase length (eLen), number of entity words in each entity phrase (eNum), entity density (eDen), and entity label consistency (elCon) values of an entity word.

To construct the macrofactor datasets, we computed metrics such as *sLen*, *eLen*, *eNum*, *eDen*, and *elCon* for each entity word within each entity type across the Revised JNLPBA, BC5CDR, and AnatEM datasets. These metrics quantify specific linguistic and structural attributes of the datasets. In addition, we included *tEWC* as a separate column to represent the total count of entity words for every kind across the datasets, quantifying the overall entity word volume. Figure 2 shows the systematic computation of these metrics except *tEWC* to augment the macrofactor datasets.

**Figure 3:**
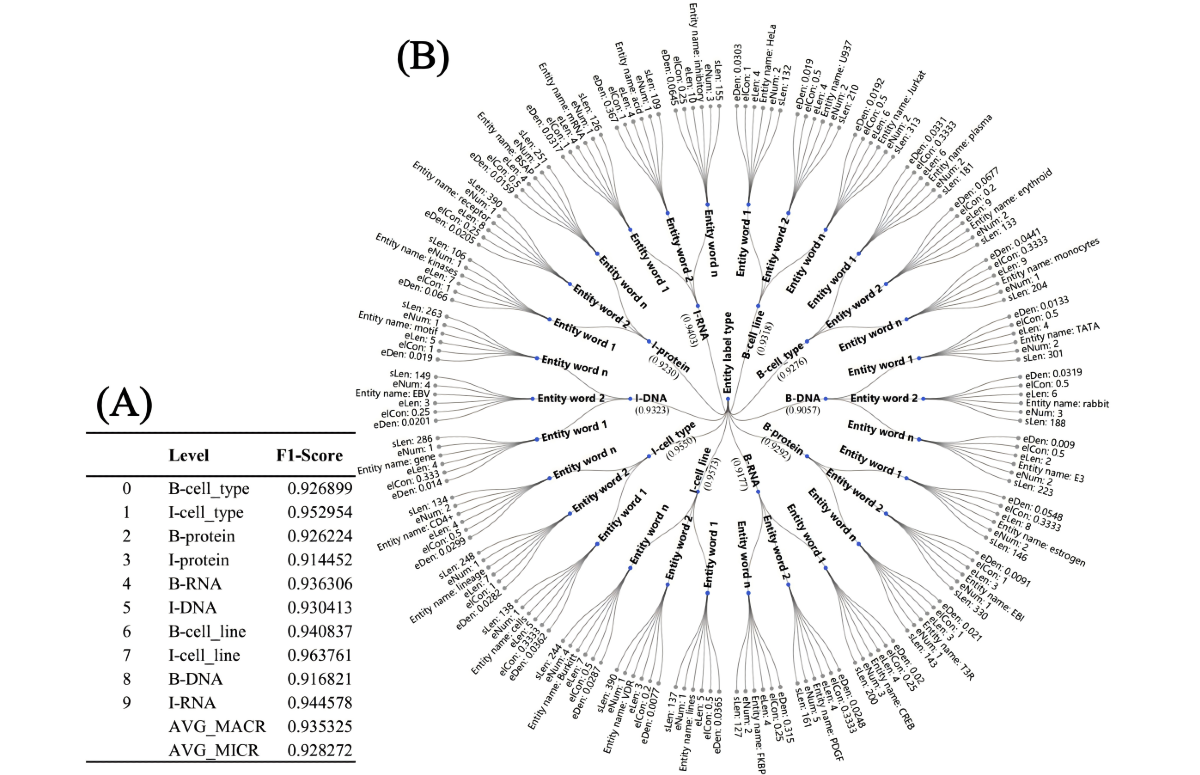
An example of 2 parts illustrating different components of each macrofactor dataset. Panel (A) displays the labels used in the datasets, whereas panel (B) presents a visualization of the macrofactor metrics—sentence length, entity phrase length, number of entity words in each entity phrase, entity density, and entity label consistency—using the radial tree layout algorithm. This visualization offers a structured view of the interrelationships among these metrics. AVG_MACRO: macroaverage; AVG_MICRO: microaverage.

Due to the supervised nature of these datasets, labels were assigned based on the *F*_1_-scores achieved by the top-performing models within 3 distinct categories: statistical machine learning models (HMM and CRF), deep learning NLP models based on BERT architecture (RoBERTa, BigBird, DeBERTa, and BioBERT), and LLMs (Gemma). The model with the highest AVG_MICRO and AVG_MACRO scores was identified for each model category. The *F*_1_-scores for each entity type, as achieved by these best-performing models, were then used as labels for the datasets. This method ensures that the labels accurately reflect the prediction accuracy for each entity type. Consequently, 9 unique macrofactor datasets were generated, each representing a combination of the computed metrics and the derived labels. Figure 3A illustrates the use of these *F*_1_-scores as labels, whereas Figure 3B provides an example of how the dataset structure was revised based on these macrofactors.

##### Preliminary Macrofactor Evaluation

Our preliminary analysis adopted a comprehensive method to assess the interactions between prediction accuracy for various entity types (eg, disease, DNA, and RNA) and 6 macrofactors. Initially, we extracted prediction accuracy data for each entity type from these NER models (HMM, CRF, RoBERTa, BigBird, DeBERTa, BioBERT, and Gemma), and we calculated the average values for macrofactor metrics such as *sLen*, *eLen*, *eNum*, *eDen*, *elCon*, and *tEWC*. We then explored the impact of each metric on the prediction accuracy for each entity type. We considered trend analysis to provide deeper insights into the relationships between these metrics and accuracy levels. In addition, the visualization of multimodel predictive trends offered a comprehensive view of model robustness across different entity types.

##### In-Depth Macrofactor Selection

###### Overview

Our subsequent research focused on identifying which macrofactors significantly impacted the models’ prediction accuracy. We developed an MFE algorithm and conducted macrofactor selection. MFE is an improved algorithm based on recursive feature elimination [49] divided into 3 layers.

###### First Layer: Factor Ranking and Selection

The MFE algorithm inputs the calculated values for *sLen*, *eLen*, *eNum*, *eDen,* *tEWC*, and *elCon* for each entity word.

Linear correlation analysis is performed for each factor using the following function. The correlation coefficient (*r*) is a statistical measure used to quantify the strength and direction of the relationship between 2 variables. *x_i_* represents individual macrofactor measurements, is the mean of all macrofactor measurements, *y_i_* represents individual model performance measurements, and is the mean of all model performance measurements. This calculation quantifies the relationship between each macrofactor and the model’s prediction accuracy.



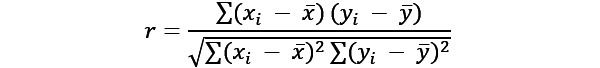



Using a method similar to the Pearson correlation method [50], factors are ranked by their correlation scores, and the top 4 are retained for further analysis. This step ensures that only the factors with the highest potential impact are advanced, thereby efficiently streamlining the feature space.

###### Second Layer: Random Forest Evaluation

A random forest model performs multiple training iterations using the selected macrofactors from the first layer.

After each training session, cross-validation is used to evaluate model performance. The macrofactor with the lowest feature importance score, indicating minimal contribution to prediction accuracy, is systematically excluded from subsequent analyses. This iterative refinement process prioritizes factors that consistently enhance model accuracy [51].

###### Third Layer: Regression Model Optimization

The refined set of macrofactors is integrated into a regression model.

The least impactful macrofactors are sequentially eliminated based on the influence of their coefficients on the model, which is calculated as follows:

Coefficient influence = β **(2)**

β is the coefficient related to the macrofactors; the model is retrained after each removal, continuing until no further improvement in performance is detected. This final step ensures that only the most impactful factors are retained, optimizing the model’s efficiency and effectiveness.

### Ethical Considerations

Ethics approval was obtained from the Institute of Medical Information and Library, Chinese Academy of Medical Sciences and Peking Union Medical College (ethics approval code: IMICAMS/01/22/HREC). After obtaining ethics approval, the Institute of Medical Information and Library, Chinese Academy of Medical Sciences and Peking Union Medical College, wrote an official ethics approval statement.

All medical NER datasets used in this paper are public datasets; no personal or sensitive information was collected, and the datasets complied with local institutional guidelines and legislation. It was unnecessary to obtain written or verbal informed consent from the participants. The experimental protocols and datasets in this study were approved by the Institute of Medical Information and Library, Chinese Academy of Medical Sciences and Peking Union Medical College. All methods were performed according to the relevant guidelines and regulations.

## Results

### Model Prediction Results

The predicted outcomes of the NER models following extensive training, testing, and hyperparameter optimization are detailed in Table 3. The statistical machine learning models, HMM and CRF, exhibited moderate performance. HMM showed relatively lower scores across all datasets, with AVG_MICRO scores of 0.7265, 0.825, and 0.6112 for the Revised JNLPBA, BC5CDR, and AnatEM datasets, respectively. Similarly, its AVG_MACRO was also lower, indicating limited effectiveness in capturing diverse entity types. On the other hand, the CRF model performed better than HMM, achieving higher AVG_MICRO scores of 0.8476, 0.9019, and 0.743 for the same datasets, respectively. The corresponding AVG_MACRO for CRF was also higher, reflecting its better overall accuracy and balance in entity recognition.

In contrast, the BERT-based deep learning models (BioBERT, RoBERTa, BigBird, and DeBERTa) consistently demonstrated a strong performance across multiple datasets. Notably, BioBERT achieved the highest results on the Revised JNLPBA and AnatEM datasets, with an AVG_MICRO of 0.932 and an AVG_MACRO of 0.9298 on the Revised JNLPBA dataset and an AVG_MICRO of 0.8494 and an AVG_MACRO of 0.6975 on the AnatEM dataset. However, on the BC5CDR dataset, these models performed slightly worse, with AVG_MICRO and AVG_MACRO values generally below 0.8726 and 0.858, respectively. This indicated that, while BERT-based models exhibited robust performance in specific contexts, their generalization capabilities varied depending on the dataset characteristics.

The Gemma model demonstrated a strong performance on the BC5CDR dataset, achieving the highest AVG_MICRO of 0.9962 and an AVG_MACRO of 0.981. However, its results were less pronounced on the Revised JNLPBA and AnatEM datasets compared to BioBERT and other models.

**Table 3:** Model prediction accuracy comparison.

Model			Revised JNLPBA^a^ dataset				BC5CDR^b^ dataset					AnatEM^c^ dataset		
			AVG_MICRO^d^		AVG_MACRO^e^		AVG_MICRO		AVG_MACRO		AVG_MICRO			AVG_MACRO
**Statistical machine learning models**														
	HMM^f^	0.7265		0.6815		0.8250		0.7002		0.6112			0.5013	
	CRF^g^	0.8476		0.8258		0.9019		0.8883		0.7430			0.5114	
**Deep learning NLP^h^ models based on BERT^i^ architecture**														
	BioBERT^j^	0.932		0.9298		0.8726		0.858		0.8494			0.6975	
	RoBERTa^k^	0.9133		0.9133		0.8313		0.8152		0.8201			0.6501	
	BigBird^l^	0.9277		0.9218		0.8461		0.8321		0.8147			0.6451	
	DeBERTa^m^	0.9256		0.921		0.8471		0.8335		0.806			0.6131	
**Large language model**														
	Gemma	0.9088		0.8298		0.9962		0.9810		0.8029			0.6496	

^a^JNLPBA: Joint Workshop on Natural Language Processing in Biomedicine and its Applications.

^b^BC5CDR: BioCreative V CDR.

^c^AnatEM: Anatomical Entity Mention.

^d^AVG_MICRO: microaverage.

^e^AVG_MACRO: macroaverage.

^f^HMM: hidden Markov model.

^g^CRF: conditional random fields.

^h^NLP: natural language processing.

^i^BERT: Bidirectional Encoder Representations From Transformers.

^j^BioBERT: Bidirectional Encoder Representations from Transformers for Biomedical Text Mining.

^k^RoBERTa: Robustly Optimized BERT Pretraining Approach.

^l^BigBird: Big Transformer Models for Efficient Long-Sequence Attention.

^m^DeBERTa: Decoding-enhanced BERT with Disentangled Attention.

**Figure 4:**
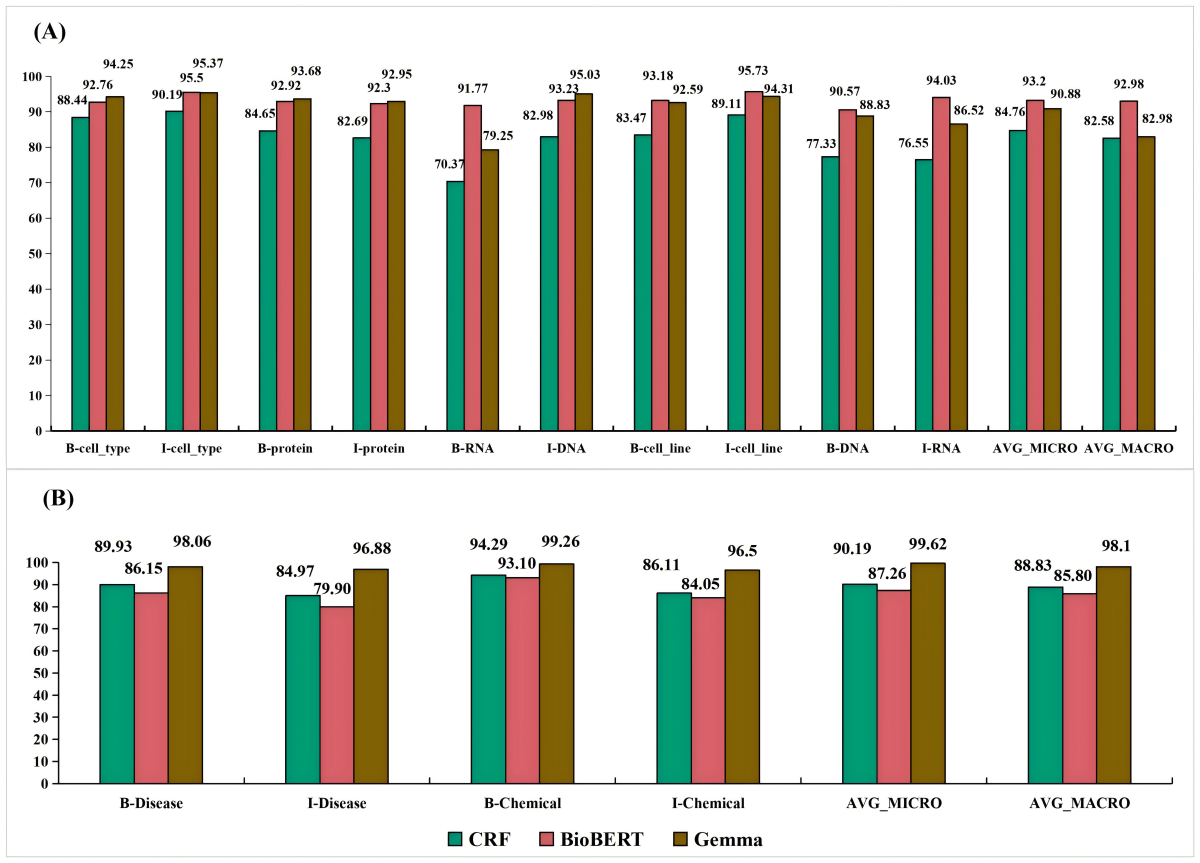
Prediction accuracy of various models on two datasets. (A) The figure shows the prediction accuracy on the Revised Joint Workshop on Natural Language Processing in Biomedicine and its Applications (JNLPBA) dataset. (B) The figure shows the prediction accuracy on the BioCreative V CDR (BC5CDR) dataset. AVG_MACRO: macroaverage; AVG_MICRO: microaverage; BioBERT: Bidirectional Encoder Representations from Transformers for Biomedical Text Mining; CRF: conditional random fields.

**Figure 5:**
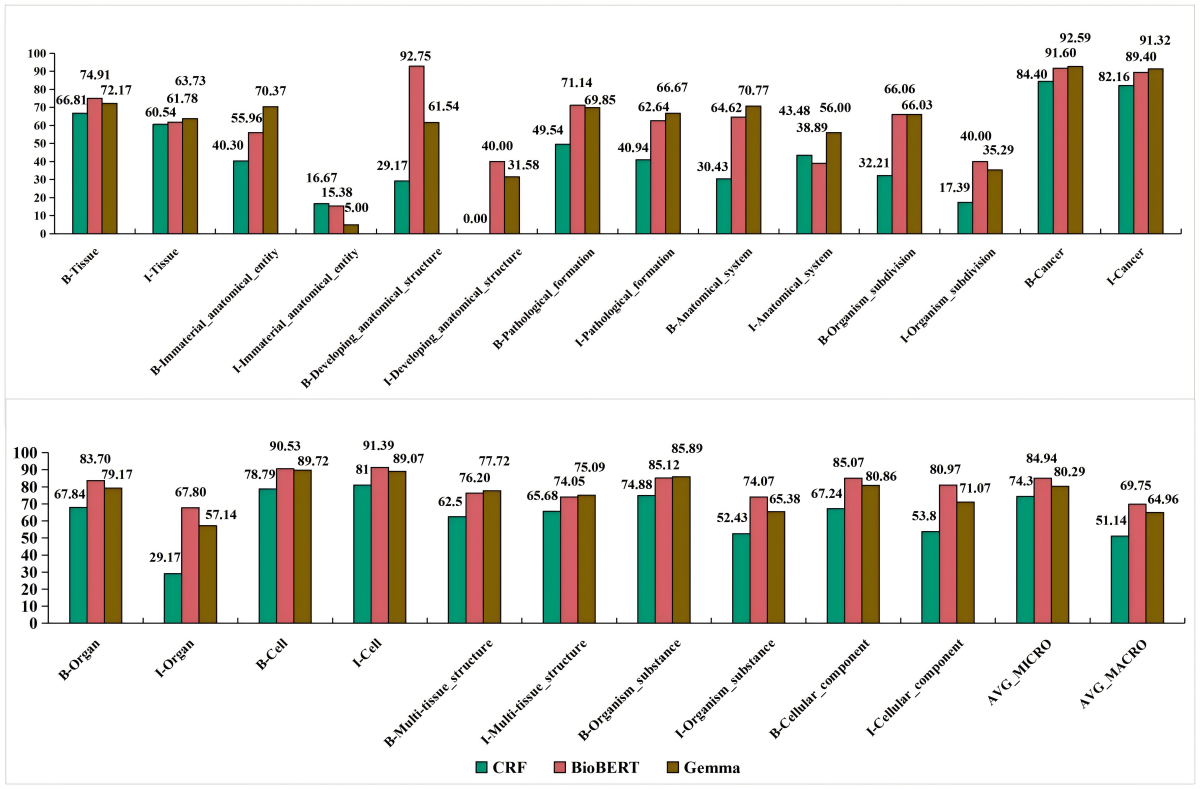
Prediction accuracy of various models on the Anatomical Entity Mention (AnatEM) dataset. AVG_MACRO: macroaverage; AVG_MICRO: microaverage; BioBERT: Bidirectional Encoder Representations from Transformers for Biomedical Text Mining; CRF: conditional random fields.

In addition, Figures 4 and 5 highlight the *F*_1_-scores for each entity type achieved by the best-performing models in each category: statistical machine learning models (represented by CRF), deep learning NLP models based on BERT architecture (represented by BioBERT), and the Gemma LLM.

The performance of the CRF, BioBERT, and Gemma models on the Revised JNLPBA dataset showed distinct differences across various entity types. BioBERT consistently outperformed CRF and Gemma across most entity types. For instance, in the “B-DNA” entity type, BioBERT attained the highest score of 0.9057 compared to CRF’s 0.7733 and Gemma’s 0.8883. Gemma also demonstrated a high performance in several entity types, such as “B-protein” (0.9368) and “I-DNA” (0.9503). Nonetheless, it showed variability in other entity types, such as “B-RNA” (0.7925), where BioBERT achieved a significantly higher score (0.9177). While showing promising results in entity types such as “I-cell_line” (0.8911), the CRF model generally performed lower than BioBERT and Gemma. Notably, CRF’s performance in the “I-RNA” entity type (0.7655) was significantly lower than that of both BioBERT (0.9403) and Gemma (0.8652).

In the BC5CDR dataset, Gemma consistently outperformed CRF and BioBERT across most entity types. For instance, in the “B-Disease” entity type, Gemma achieved an *F*_1_-score of 0.9806, significantly higher than that of CRF (0.8993) and BioBERT (0.8615). Similarly, in the “I-Disease” entity type, Gemma attained the highest score of 0.9688 compared to CRF’s 0.8497 and BioBERT’s 0.7990. Gemma also demonstrated exceptional performance in the “B-Chemical” entity type (0.9926), surpassing CRF (0.9429) and BioBERT (0.9310). The “I-Chemical” entity type showed similar trends, with Gemma achieving an *F*_1_-score of 0.9650, higher than that of CRF (0.8611) and BioBERT (0.8405). The CRF model exhibited moderate performance, achieving lower *F*_1_-scores than Gemma but often outperforming BioBERT, particularly in the “B-Disease” and “B-Chemical” entity types.

In the AnatEM dataset, BioBERT consistently outperformed CRF and Gemma across most entity types. For instance, in the “B-Cell” entity type, BioBERT achieved an *F*_1_-score of 0.9053, higher than that of both CRF (0.7879) and Gemma (0.8972). Similarly, in the “I-Cell” entity type, BioBERT attained the highest score of 0.9139 compared to CRF’s 0.81 and Gemma’s 0.8907. Gemma, while showing high performance in specific entity types such as “B-Cancer” (0.9259) and “I-Cancer” (0.9132), demonstrated more variability across different entity types. For example, Gemma’s performance in the “I-Organ” entity type was lower (0.5714) than BioBERT’s (0.6780). Although the CRF model showed promising results in specific entity types such as “B-Cancer” (0.844), it generally performed lower than BioBERT and Gemma. Mainly, CRF’s performance in types such as “I-Organ” (0.2917) and “I-Developing_anatomical_structure” (0.00) was significantly lower than that of both BioBERT and Gemma.

### Fine-Tuning Results of the BERT-Based Models

**Figure 6:**
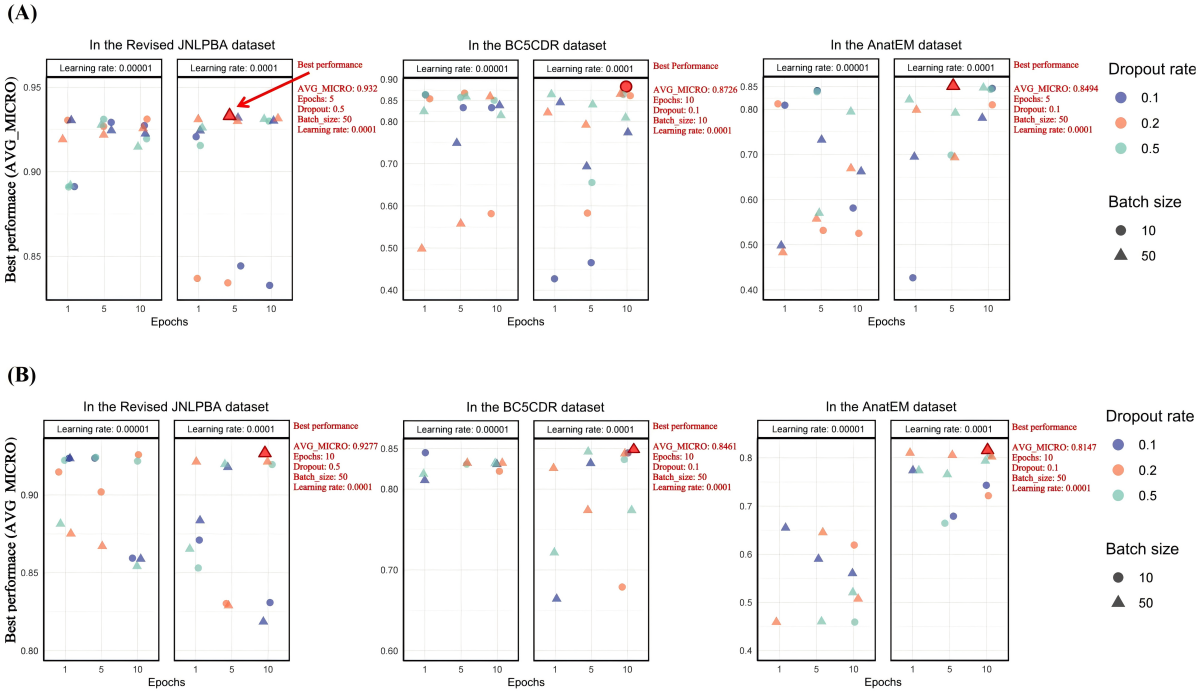
Fine-tuning results of Bidirectional Encoder Representations from Transformers (BioBERT) and Big Transformer Models for Efficient Long-Sequence Attention (BigBird)—microaverage (AVG_MICRO) scores across datasets. (A) The figure shows AVG_MICRO scores for BioBERT across datasets. (B) The figure shows AVG_MICRO scores for BigBird across datasets. AVG_MICRO is used as the sole leveraged metric because both the AVG_MICRO and macroaverage metrics exhibit similar trends, generally increasing or decreasing. This similarity indicates that using AVG_MICRO alone is sufficient to understand the overall performance of the models, making the results more straightforward and focused. AnatEM: Anatomical Entity Mention; BC5CDR: BioCreative V CDR; BERT: Bidirectional Encoder Representations From Transformers; JNLPBA: Joint Workshop on Natural Language Processing in Biomedicine and its Applications.

**Figure 7:**
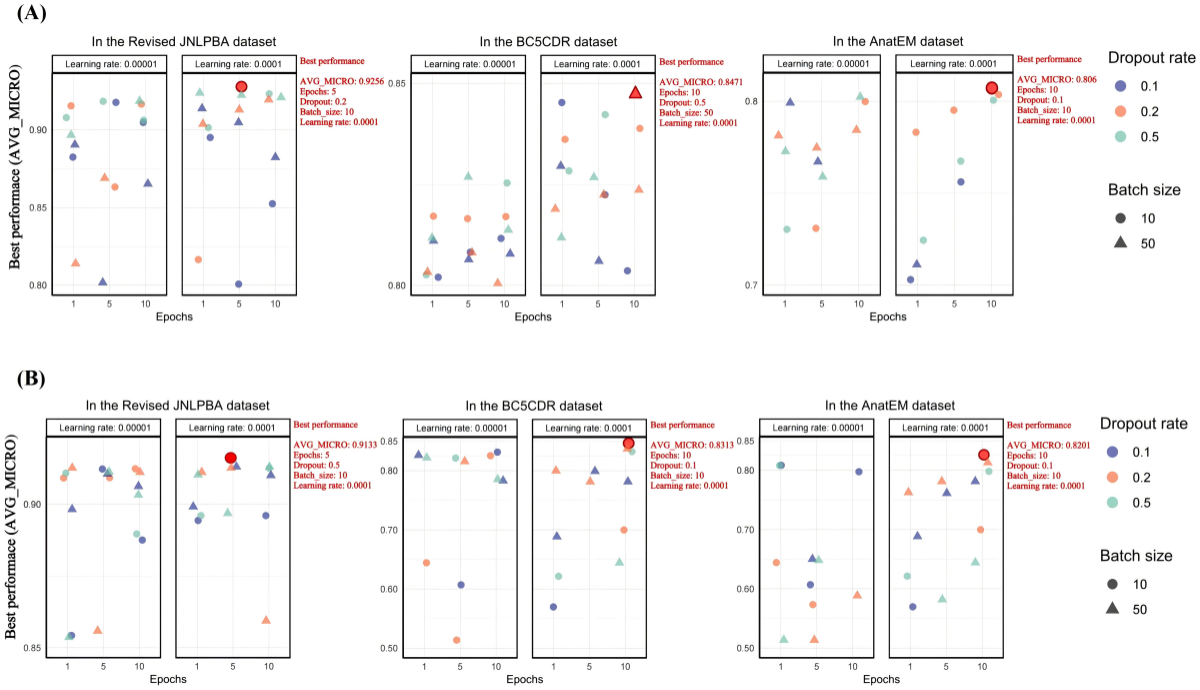
Fine-tuning results of the Decoding-enhanced Bidirectional Encoder Representations from Transformers (BERT) with Disentangled Attention (DeBERTa) and Robustly Optimized BERT Pretraining Approach (RoBERTa) models—microaverage (AVG_MICRO) scores across datasets. (A) The figure shows AVG_MICRO scores for DeBERTa across datasets. (B) The figure shows AVG_MICRO scores for RoBERTa across datasets. AnatEM: Anatomical Entity Mention; BC5CDR: BioCreative V CDR; JNLPBA: Joint Workshop on Natural Language Processing in Biomedicine and its Applications.

On the basis of the observations from Figures 6 and 7, the fine-tuning of models based on BERT architecture (BioBERT, BigBird, DeBERTa, and RoBERTa) across the Revised JNLPBA, BC5CDR, and AnatEM datasets demonstrated that a learning rate of 0.0001 consistently yielded the best prediction accuracy. However, the optimal configurations for batch size, epochs, and dropout rate varied significantly among the models and datasets.

BioBERT achieved an AVG_MICRO of 0.932 on the Revised JNLPBA dataset with a batch size of 50, a dropout rate of 0.5, and 5 epochs. On the BC5CDR dataset, it reached an AVG_MICRO of 0.8726 with a lower dropout rate of 0.1, a batch size of 10, and 10 epochs, whereas on the AnatEM dataset, an AVG_MICRO of 0.8494 was obtained with a dropout rate of 0.1, a batch size of 50, and 5 epochs.

BigBird consistently performed best with a batch size of 50 and 10 epochs, achieving an AVG_MICRO of 0.9277 on the Revised JNLPBA dataset. It also showed a strong performance on the BC5CDR and AnatEM datasets, with AVG_MICRO scores of 0.8461 and 0.8147, respectively.

DeBERTa recorded an AVG_MICRO of 0.9256 on the Revised JNLPBA dataset with a batch size of 10, a dropout rate of 0.2, and 5 epochs. On the BC5CDR dataset, it achieved an AVG_MICRO of 0.8471 with a higher dropout rate of 0.5, a batch size of 50, and 10 epochs. For the AnatEM dataset, an AVG_MICRO of 0.806 was achieved with a dropout rate of 0.1, a batch size of 10, and 10 epochs.

RoBERTa demonstrated optimal performance across the 3 datasets using a batch size of 10. On the Revised JNLPBA dataset, RoBERTa achieved an AVG_MICRO of 0.9313 with a dropout rate of 0.5 and 5 epochs. On the BC5CDR dataset, it obtained an AVG_MICRO of 0.8313 with a dropout rate of 0.1 and 10 epochs. Similarly, on the AnatEM dataset, RoBERTa achieved an AVG_MICRO of 0.8201 with a dropout rate of 0.1 and 10 epochs.

These results underscore the critical role of hyperparameter tuning as each model and dataset required specific configurations to achieve optimal performance. While a consistent learning rate of 0.0001 was effective across all models, batch size, epoch, and dropout rate variations were necessary to adapt to the specific characteristics of different datasets and model architectures.

### Fine-Tuning Results of the Gemma Model

Table 4 shows significant improvements in the Gemma model’s performance metrics after fine-tuning using the LoRA technique across various datasets. The AVG_MICRO metric increased by 0.0245 for the Revised JNLPBA dataset, by 0.0111 for the BC5CDR dataset, and by 0.0083 for the AnatEM dataset. Similarly, the AVG_MACRO metric increased by 0.0173 for the Revised JNLPBA dataset, decreased by 0.0027 for the BC5CDR dataset, and increased by 0.0192 for the AnatEM dataset.

**Table 4:** Comparison of prediction accuracy (as microaverage [AVG_MICRO] and macroaverage [AVG_MACRO] metrics) for the Gemma model before and after fine-tuning on different datasets.

Model, dataset, and leveraged metrics		Before fine-tuning	After fine-tuning
**Revised JNLPBA^a^**			
	AVG_MICRO	0.8843	0.9088
	AVG_MACRO	0.8125	0.8298
**BC5CDR^b^**			
	AVG_MICRO	0.9851	0.9962
	AVG_MACRO	0.9837	0.9810
**AnatEM^c^**			
	AVG_MICRO	0.7946	0.8029
	AVG_MACRO	0.6304	0.6496

^a^JNLPBA:Joint Workshop on Natural Language Processing in Biomedicine and its Applications.

^b^BC5CDR: BioCreative V CDR.

^c^AnatEM: Anatomical Entity Mention.

### Model Resource Use Results

### Overview

**Figure 8:**
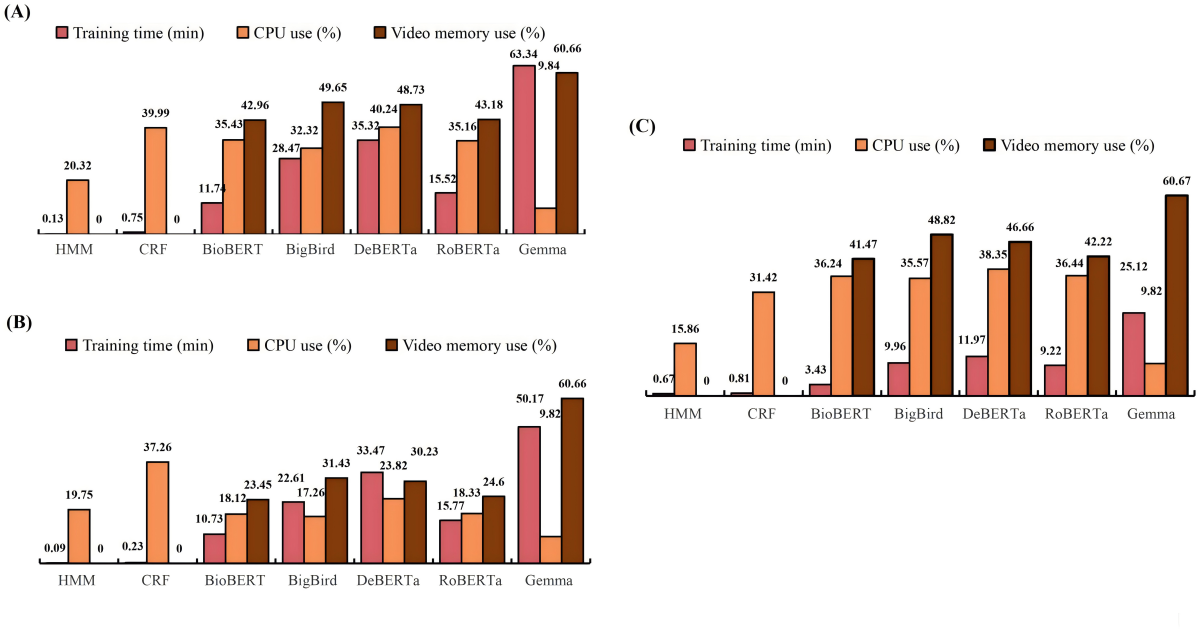
Training time and central processing unit (CPU) and graphics processing unit (GPU) uses of the models. (A) The figure shows the resource use results on the Revised Joint Workshop on Natural Language Processing in Biomedicine and its Applications (JNLPBA) dataset. (B) The figure shows the resource use results on the BioCreative V CDR (BC5CDR) dataset. (C) The figure shows the resource use results on the Anatomical Entity Mention (AnatEM) dataset. BERT: Bidirectional Encoder Representations from Transformers; BioBERT: Bidirectional Encoder Representations from Transformers for Biomedical Text Mining; CRF: conditional random fields; DeBERTa: Decoding-enhanced BERT with Disentangled Attention; HMM: hidden Markov model; RoBERTa: Robustly Optimized BERT Pretraining Approach; BigBird: Big Transformer Models for Efficient Long-Sequence Attention.

In evaluating prediction accuracy among the HMM, CRF, RoBERTa, BigBird, DeBERTa, BioBERT, and Gemma models, we documented their training time, CPU use, and GPU memory consumption performance, as shown in Figure 8.

#### Training Time

Gemma consistently required the most extended training times across all datasets, reaching a peak of 63.34 minutes in the Revised JNLPBA dataset. In stark contrast, HMM was notably more efficient, completing training in only 0.13 minutes in the same dataset and a mere 0.09 minutes in the BC5CDR dataset.

#### CPU Use

DeBERTa recorded the highest CPU use—40.24% and 38.35% in the Revised JNLPBA and AnatEM datasets, respectively. In the BC5CDR dataset, CRF had the highest CPU use at 37.26%. In contrast, Gemma demonstrated minimal CPU requirements, with use rates consistently at approximately 10% across all 3 datasets.

#### GPU Memory Consumption

Gemma had the highest GPU use, with rates consistently at approximately 61% across all 3 datasets. In contrast, the HMM and CRF traditional machine learning models recorded zero GPU use. This is because these models primarily rely on the CPU for their computations and do not leverage the parallel processing capabilities of GPUs, which are designed to accelerate deep learning tasks. Among the BERT-based models, BioBERT exhibited the lowest GPU use, consuming 42.96% and 41.47% in the Revised JNLPBA and AnatEM datasets, respectively, and only 23.45% in the BC5CDR dataset. This lower GPU consumption indicates that BioBERT, while still using GPU resources, does so more efficiently than Gemma, potentially due to its optimized architecture and more efficient use of GPU memory for processing.

#### Overall

Gemma required significant resources and was characterized by the highest GPU use across all datasets and the most extended training times. This computational intensity may enhance accuracy but comes at the cost of increased operational resources. In contrast, BioBERT demonstrated high prediction accuracy and lower resource consumption among the deep learning models, indicating its efficiency and suitability for environments with strict resource constraints.

### Entity Type Prediction Accuracy Results

**Figure 9:**
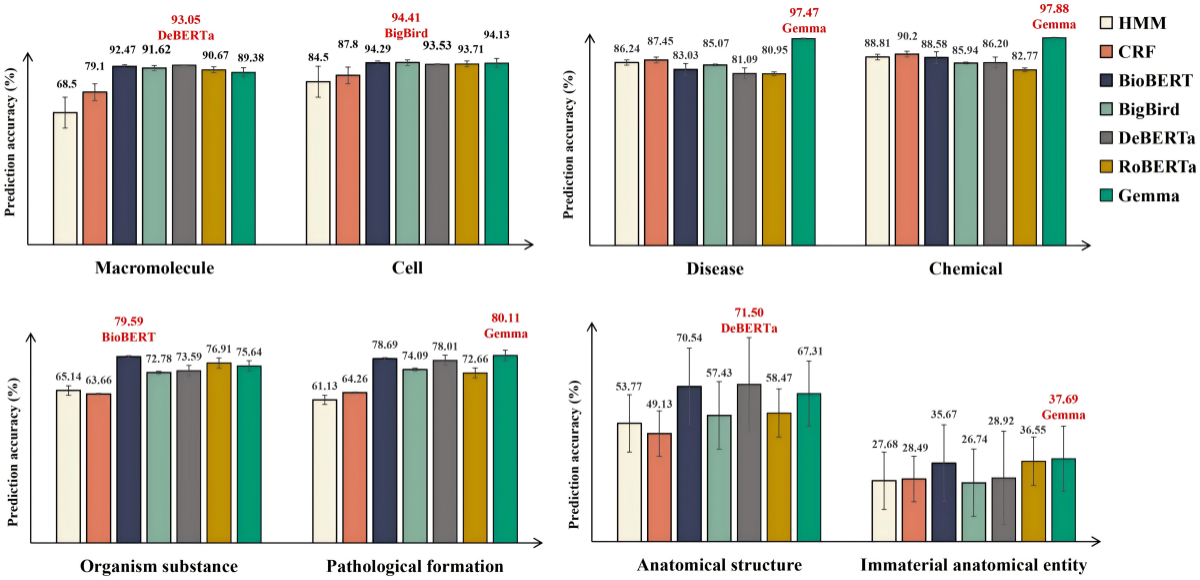
Relationship between the models’ prediction accuracy and merged entity types. BERT: Bidirectional Encoder Representations from Transformers; BioBERT: Bidirectional Encoder Representations from Transformers for Biomedical Text Mining; CRF: conditional random fields; DeBERTa: Decoding-enhanced BERT with Disentangled Attention; HMM: hidden Markov model; RoBERTa: Robustly Optimized BERT Pretraining Approach; BigBird: Big Transformer Models for Efficient Long-Sequence Attention.

On the basis of the data presented in Figure 9, we identified specific strengths in different models using a categorical relaxation method to merge entity types. Gemma achieved the highest prediction accuracy in the “chemical,” “disease,” “pathological formation,” and “immaterial anatomical entity” types, whereas BioBERT excelled in the “organism substance” entity type. DeBERTa performed best in the “macromolecule” and “anatomical structure” entity types, demonstrating its strengths. In addition, BigBird showed a superior performance in the “cell” type.

### Macrofactor Trends Impacting Model Prediction Accuracy

The results of this section primarily involve the trend relationships between the prediction accuracy of 7 NER models and 6 macrofactor metrics, focusing on the average values of these metrics across various datasets.

**Figure 10:**
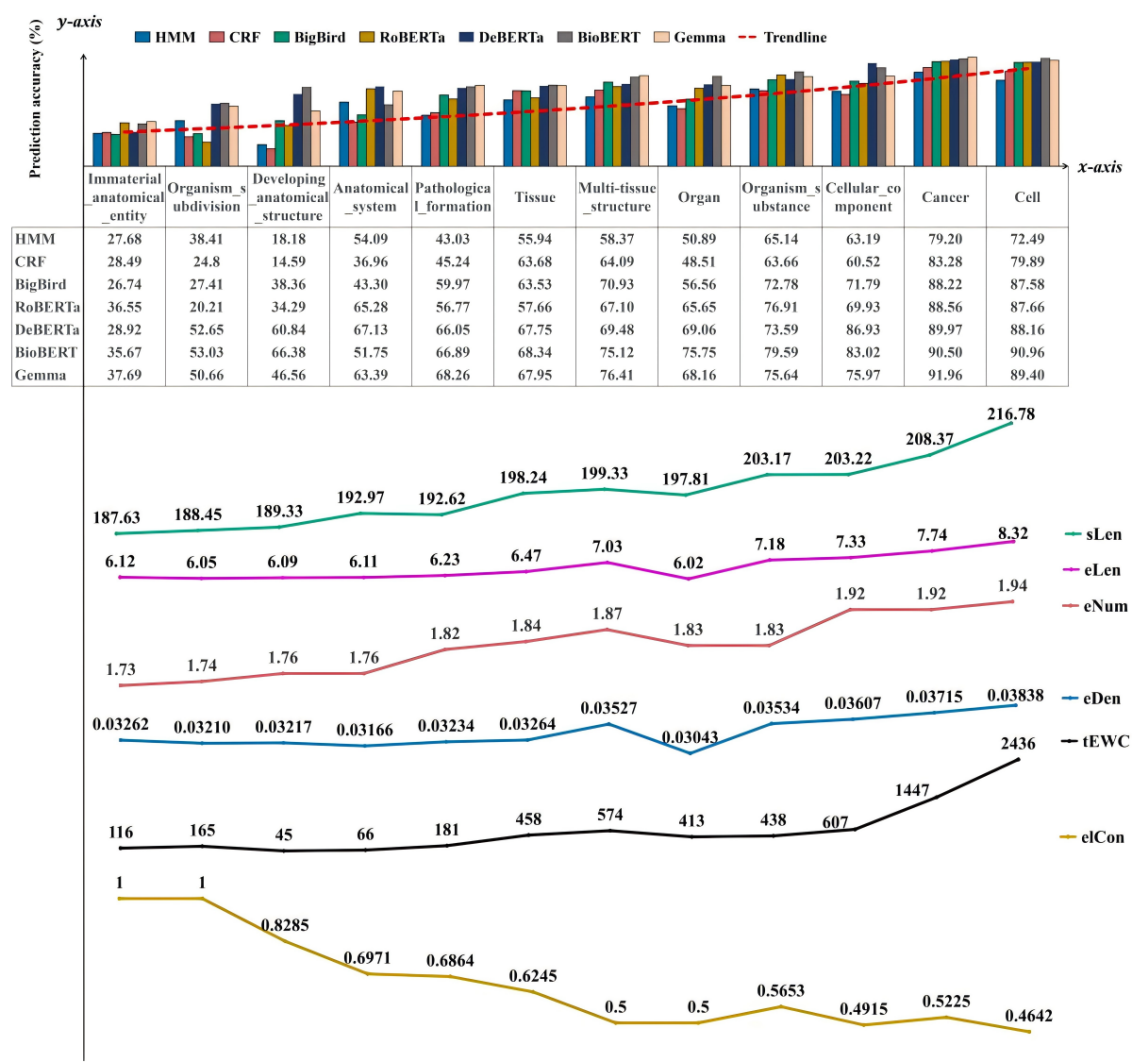
Relationship between macrofactors and model prediction accuracy in the Anatomical Entity Mention (AnatEM) dataset. BERT: Bidirectional Encoder Representations from Transformers; BigBird: Big Transformer Models for Efficient Long-Sequence Attention; BioBERT: Bidirectional Encoder Representations from Transformers for Biomedical Text Mining; CRF: conditional random fields; DeBERTa: Decoding-enhanced BERT with Disentangled Attention; eDen: entity density; elCon: entity label consistency; eLen: entity phrase length; eNum: number of entity words in each entity phrase; HMM: hidden Markov model; RoBERTa: Robustly Optimized BERT Pretraining Approach; sLen: sentence length; tEWC: total entity word count in each entity type.

In the AnatEM dataset, as depicted in Figure 10, there was a distinct correlation—entity types that yielded higher prediction accuracies corresponded to increased values in *sLen*, *eLen*, *eNum*, *eDen,* and *tEWC*. This pattern indicates that the model’s ability to accurately predict entities is enhanced with the rising complexity and volume of data associated with those entity types. Conversely, an inverse relationship was observed with *elCon*, which decreased as the other metrics increased. For instance, BioBERT recorded a high prediction accuracy of 90.96% in the “cell” entity type. This outstanding performance correlated with the highest metrics observed in the dataset—*sLen* peaked at 216.78, *eLen* peaked at 8.32, *eNum* peaked at 1.94, and *eDen* peaked at 0.03838. Then, the “cell” entity type’s *tEWC* was notably high at 2436, demonstrating the BioBERT’s ability to process extensive textual data effectively. However, *elCon* was significantly lower at 0.4642. This indicates that, although this model is adept at managing complex and voluminous data, it does not consistently ensure accurate entity labeling, suggesting a trend in which higher accuracy and complexity metrics do not correspond with label consistency.

**Figure 11:**
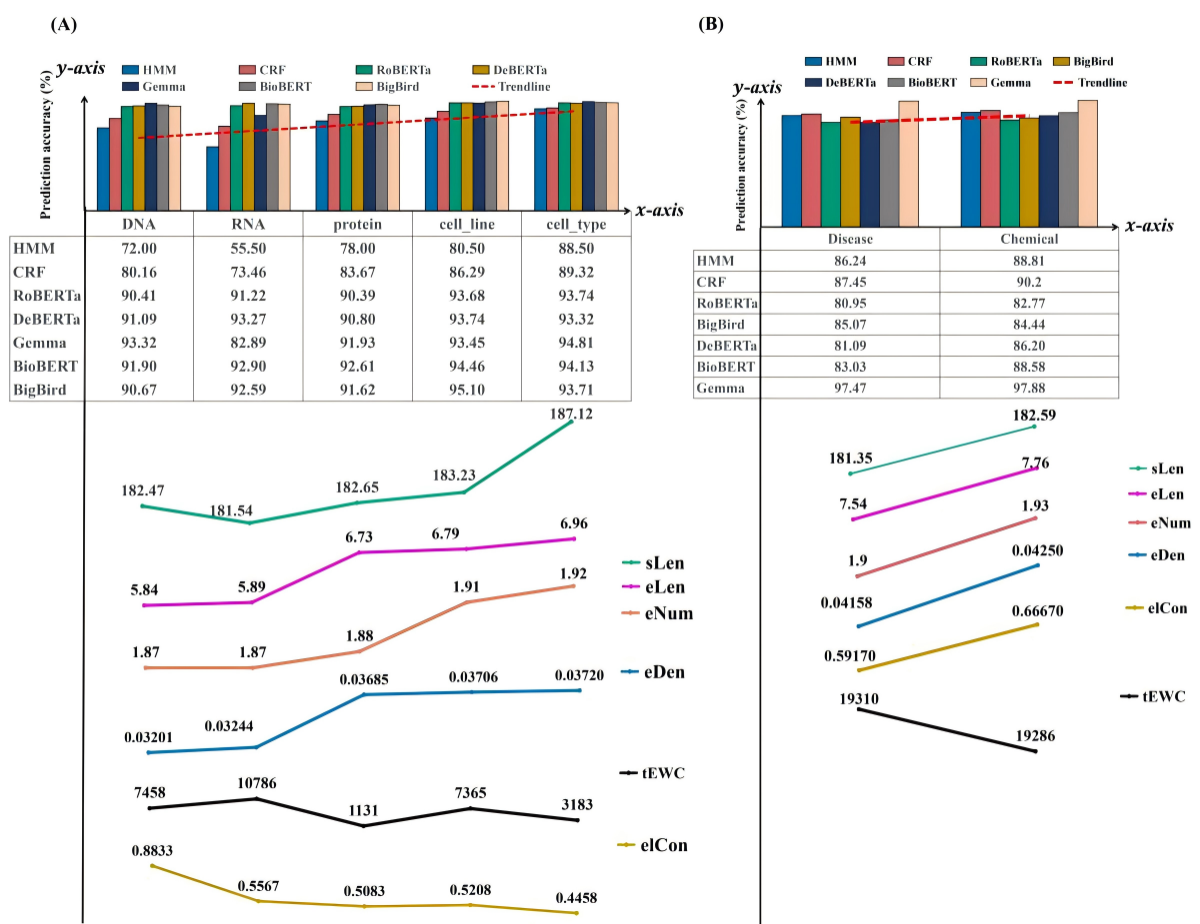
Relationship between macrofactors and model prediction accuracy in two datasets. (A) The figure shows the relationship in the Revised Joint Workshop on Natural Language Processing in Biomedicine and its Applications (JNLPBA) dataset. (B) The figure shows the relationship in the BioCreative V CDR (BC5CDR) dataset. BERT: Bidirectional Encoder Representations from Transformers; BioBERT: Bidirectional Encoder Representations from Transformers for Biomedical Text Mining; CRF: conditional random fields; DeBERTa: Decoding-enhanced BERT with Disentangled Attention; eDen: entity density; elCon: entity label consistency; eLen: entity phrase length; eNum: number of entity words in each entity phrase; HMM: hidden Markov model; RoBERTa:Robustly Optimized BERT Pretraining Approach; sLen: sentence length; tEWC: total entity word count in each entity type; BigBird: Big Transformer Models for Efficient Long-Sequence Attention.

The Revised JNLPBA dataset substantiated these observations, as shown in Figure 11. The “cell_type” entity type generally yielded a higher accuracy among the 7 models, showing elevated values for *sLen*, *eLen*, *eNum*, and *eDen*, with average values recorded at 187.12 (SD 10.52), 7.76 (SD 1.24), 1.91 (SD 0.32), and 0.0372 (SD 0.005), respectively. This indicated that the models achieved high accuracy and effectively handled denser entity distributions. Moreover, there seemed to be a negative correlation between higher values of *elCon* and *tEWC* and these accuracies. In addition, a comparable trend emerged in the BC5CDR dataset, where the “chemical” entity type yielded the highest accuracy across the RoBERTa, DeBERTa, and BioBERT models, aligning with the highest measurements of *sLen*, *eLen*, *eNum*, *eDen*, and *tEWC*, coupled with the lowest value of *elCon*.

### Macrofactor Selection Results

Examining Tables 5-7, the results from the MFE algorithm reveal the importance of different macrofactors across various models and datasets. In the CRF model, *eNum* was identified as the most influential macrofactor for prediction accuracy in the Revised JNLPBA and AnatEM datasets. In contrast, *eLen* was found to be more influential in the BC5CDR dataset. This finding indicates that the *eNum* and *eLen* play varying roles depending on the dataset’s characteristics. The Gemma model exhibited results similar to those of the CRF model, with *eNum* significantly impacting prediction accuracy for the Revised JNLPBA and AnatEM datasets. At the same time, *eLen* significantly influenced prediction accuracy for the BC5CDR dataset.

In the BioBERT model, *eNum* consistently emerged as the most critical macrofactor across all datasets (Revised JNLPBA, BC5CDR, and AnatEM); despite varying macrofactor combinations in the initial 2 layers, *eNum* was consistently chosen for the final layer. This indicates that, among the 6 macrofactors, *eNum* significantly influences BioBERT’s prediction accuracy.

**Table 5:** Macrofactors selected by the multilevel factor elimination (MFE) algorithm in the conditional random fields model across different datasets.

MFE algorithm	On the basis of the Revised JNLPBA^a^ dataset	On the basis of the BC5CDR^b^ dataset	On the basis of the AnatEM^c^ dataset
Input	sLen^d^, eLen^e^, eNum^f^, eDen^g^, elCon^h^, and tEWC^i^	sLen, eLen, eNum, eDen, elCon, and tEWC	sLen, eLen, eNum, eDen, elCon, and tEWC
Layer 1	sLen, eLen, eNum, and elCon	eLen, eNum, eDen, and tEWC	sLen, eLen, eNum, and eDen
Layer 2	sLen and eNum	eLen and eNum	eNum and eDen
Layer 3	eNum	eLen	eNum

^a^JNLPBA: Joint Workshop on Natural Language Processing in Biomedicine and its Applications.

^b^BC5CDR: BioCreative V CDR.

^c^AnatEM: Anatomical Entity Mention.

^d^sLen: sentence length.

^e^eLen: entity phrase length.

^f^eNum: number of entity words in each entity phrase.

^g^eDen: entity density.

^h^elCon: entity label consistency.

^i^tEWC: total entity word count in each entity type.

**Table 6:** Macrofactors selected by the multilevel factor elimination (MFE) algorithm in the Bidirectional Encoder Representations from Transformers for Biomedical Text Mining (BioBERT) model across different datasets.

MFE algorithm	On the basis of the Revised JNLPBA^a^ dataset	On the basis of the BC5CDR^b^ dataset	On the basis of the AnatEM^c^ dataset
Input	sLen^d^, eLen^e^, eNum^f^, eDen^g^, elCon^h^, and tEWC^i^	sLen, eLen, eNum, eDen, elCon, and tEWC	sLen, eLen, eNum, eDen, elCon, and tEWC
Layer 1	eLen, eNum, elCon, and tEWC	eLen, eNum, eDen, and tEWC	sLen, eLen, eNum, and eDen
Layer 2	eLen and eNum	eNum and tEWC	eLen and eNum
Layer 3	eNum	eNum	eNum

^a^JNLPBA: Joint Workshop on Natural Language Processing in Biomedicine and its Applications.

^b^BC5CDR: BioCreative V CDR.

^c^AnatEM: Anatomical Entity Mention.

^d^sLen: sentence length.

^e^eLen: entity phrase length.

^f^eNum: number of entity words in each entity phrase.

^g^eDen: entity density.

^h^elCon: entity label consistency.

^i^tEWC: total entity word count in each entity type.

**Table 7:** Macrofactors selected by the multilevel factor elimination (MFE) algorithm in the Gemma model across different datasets.

MFE algorithm	On the basis of the Revised JNLPBA^a^ dataset	On the basis of the BC5CDR^b^ dataset	On the basis of the AnatEM^c^ dataset
Input	sLen^d^, eLen^e^, eNum^f^, eDen^g^, elCon^h^, and tEWC^i^	sLen, eLen, eNum, eDen, elCon, and tEWC	sLen, eLen, eNum, eDen, elCon, and tEWC
Layer 1	sLen, eNum, elCon, and tEWC	eLen, eNum, eDen, and elCon	sLen, eLen, eNum, and tEWC
Layer 2	sLen and eNum	eLen and eNum	eLen and eNum
Layer 3	eNum	eLen	eNum

^a^JNLPBA: Joint Workshop on Natural Language Processing in Biomedicine and its Applications.

^b^BC5CDR: BioCreative V CDR.

^c^AnatEM: Anatomical Entity Mention.

^d^sLen: sentence length.

^e^eLen: entity phrase length.

^f^eNum: number of entity words in each entity phrase.

^g^eDen: entity density.

^h^elCon: entity label consistency.

^i^tEWC: total entity word count in each entity type.

## Discussion

### Principal Findings

This study comprehensively evaluated various NER models in medical informatics, focusing on prediction accuracy, resource use, and the impact of macrofactors and hyperparameters. The primary findings indicate that BERT-based models (BioBERT, RoBERTa, BigBird, and DeBERTa) exhibited generally higher accuracy than traditional statistical models (HMM and CRF). These BERT-based models, fine-tuned using the ADAM optimizer with a consistent learning rate of 0.0001, demonstrated outstanding performance. Among them, BioBERT excelled due to its specialized pretraining on extensive medical literature. The Gemma LLM, fine-tuned using the LoRA technique, achieved the highest accuracy on the BC5CDR dataset but showed variability across the other datasets, highlighting the need for further optimization. Macrofactors such as “entity phrase length (*eLen*)” and “the number of entity words in each entity phrase (*eNum*)” significantly influenced model performance, with the MFE algorithm effectively filtering these macrofactors. Computational resource use revealed that, while Gemma required substantial resources, BioBERT was a balanced NER model with high prediction accuracy and lower computational demands, making it suitable for resource-constrained environments. These findings underscore the importance of continuous refinement and dataset-specific optimization to advance NER capabilities in medical informatics.

### Evaluation of Predictive Performance Across NER Models

Evaluating various NER models on medical datasets provides significant insights into their strengths and limitations, highlighting the complexity of accurately identifying and categorizing medical entities. The performance disparity among statistical machine learning models, deep learning models based on BERT architecture, and the Gemma LLM is evident, with the latter 2 consistently demonstrating superior accuracy and robustness.

Statistical machine learning models such as HMM and CRF exhibited a moderate performance in medical NER tasks. The HMM’s relatively lower scores across all datasets, with AVG_MICRO scores of 0.7265, 0.825, and 0.6112 for the Revised JNLPBA, BC5CDR, and AnatEM datasets, respectively, reflected its limited capacity to capture the nuanced patterns inherent in medical texts. While CRF outperformed HMM with higher AVG_MICRO scores of 0.8476, 0.9019, and 0.743 for the same datasets, respectively, it still fell short compared to the deep learning models. This indicates inherent limitations in CRF’s feature engineering and sequence modeling capabilities, which are less adept at handling the complexity and variability of medical entities.

In contrast, the BERT-based models (BioBERT, RoBERTa, BigBird, and DeBERTa) consistently demonstrated a strong performance across multiple datasets. BioBERT’s superior performance on the Revised JNLPBA and AnatEM datasets, with AVG_MICRO scores of 0.932 and 0.8494 and AVG_MACRO scores of 0.9298 and 0.6975, respectively, can be attributed to its specialized pretraining on large-scale medical literature. This pretraining allows BioBERT to capture domain-specific nuances, enhancing its ability to recognize and classify complex medical entities accurately. In the Revised JNLPBA dataset, BioBERT achieved the highest *F*_1_-scores in categories such as “B-DNA” (0.9057) and “I-cell_type” (0.9550), showcasing its robust generalization capabilities. However, the slightly lower performance of these models on the BC5CDR dataset, where AVG_MICRO and AVG_MACRO scores were generally below 0.8726 and 0.858, respectively, suggests potential overfitting or sensitivity to the specific characteristics of this dataset. This variability underscores the necessity for further fine-tuning and incorporating additional domain-specific knowledge to improve the models’ generalization capabilities across diverse datasets.

The Gemma LLM demonstrated exceptional performance on the BC5CDR dataset, achieving the highest AVG_MICRO score of 0.9962 and an AVG_MACRO score of 0.981. In specific entity types within this dataset, Gemma attained *F*_1_-scores of 0.9806 in “B-Disease” and 0.9926 in “B-Chemical,” indicating its strong capability to recognize these entities. This suggests that Gemma’s architecture and training regimen are particularly well suited for this dataset, which contains only 2 entity types (disease and chemical). The narrow focus of the BC5CDR dataset may have allowed Gemma to optimize its performance more effectively than on more complex datasets with diverse entity types. However, Gemma’s less pronounced results on the Revised JNLPBA and AnatEM datasets, with AVG_MICRO scores of 0.9088 and 0.8029 and AVG_MACRO scores of 0.8298 and 0.6496, respectively, indicate that, while it excels in binary entity recognition, it may require further adjustments to handle more complex, multi-entity datasets effectively. In the Revised JNLPBA dataset, for instance, Gemma showed a high performance in categories such as “B-protein” (0.9368) and “I-DNA” (0.9503) but struggled in “B-RNA” (0.7925) compared to BioBERT’s 0.9177, highlighting areas for potential improvement.

**Figure 12:**
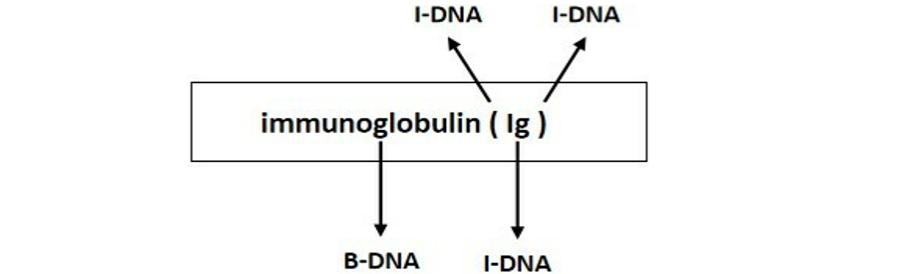
Entity type division.

Furthermore, we found significant variability in prediction accuracy across different entity types for models such as BioBERT, Gemma, and CRF, as evidenced by contrasting *F*_1_-scores for entities like ‘B-Developing_anatomical_structure’ and ‘I-Immaterial_anatomical_entity.’ This inconsistency, illustrated in Figures 4 and 5, is primarily attributed to 2 interrelated factors: uneven data distribution and the complexities inherent in medical terminologies. First, uneven data distribution and noise in datasets such as the Revised JNLPBA, BC5CDR, and AnatEM impeded the models’ ability to fully understand and accurately predict underrepresented entity types, affecting their overall performance. For example, the “protein” and “cell_type” entities in the Revised JNLPBA dataset are significantly more abundant (5256 and 2070 entity words, respectively) compared to “cell_line” and “RNA” (404 and 161 entity words, respectively). Second, multiple entity types for a single entity word complicate prediction. For instance, in the Revised JNLPBA dataset, certain symbols function as entity words within entity phrases. As depicted in Figure 12, the parentheses in the entity phrase “immunoglobulin (Ig)” from the training set are categorized as the “I-DNA” entity type. However, when predicting the entity types of parentheses in the test set, the models occasionally misclassify them as “I-DNA” even though they do not belong to this entity type in some contexts. Specifically, the phrase “(PCBA)” in the test set is not recognized as an entity phrase. However, the models may erroneously assign the parentheses to the “I-DNA” entity type, leading to prediction errors.

These findings underscore the importance of selecting and optimizing models based on the specific characteristics of the datasets to which they are applied. The consistent performance of the deep learning models, particularly those based on BERT architecture, and the promising results from LLMs such as Gemma highlight the potential for advanced NER models to improve medical entity recognition significantly. However, achieving optimal results across diverse medical contexts will require continuous refinement and adaptation of these models to the unique nuances of each dataset.

### Impact of Hyperparameters and Optimizer on the Fine-Tuning of the BERT-Based Models

The fine-tuning results of the BERT-based models (BioBERT, BigBird, DeBERTa, and RoBERTa) across the Revised JNLPBA, BC5CDR, and AnatEM datasets reveal the significant role of hyperparameter tuning and the effectiveness of the ADAM optimizer in achieving optimal prediction accuracy in medical NER tasks.

The consistent use of the ADAM optimizer with a learning rate of 0.0001 across all models demonstrated its efficacy in fine-tuning BERT-based architectures. The ADAM optimizer’s adaptive learning rate mechanism, which adjusts based on the first and second moments of the gradients, ensures efficient and effective convergence. This balance is crucial for fine-tuning complex models such as BERT and its variants as it mitigates the risk of overshooting the optimal point or converging too slowly. The success of this learning rate underscores the importance of selecting a rate that balances convergence speed and accuracy.

Beyond the learning rate, hyperparameters such as batch size, epochs, and dropout rate were critical in determining model performance. Optimal configurations for these parameters varied significantly across models and datasets, emphasizing the need for dataset-specific and model-specific tuning. For instance, BioBERT achieved an AVG_MICRO of 0.932 on the Revised JNLPBA dataset with a batch size of 50, a dropout rate of 0.5, and 5 epochs. In contrast, a smaller batch size of 10 was necessary for optimal prediction accuracy on the BC5CDR dataset. This variation illustrates how dataset characteristics heavily influence batch size selection; larger batch sizes can improve computational efficiency by leveraging the parallel processing capabilities of GPUs, whereas smaller batch sizes may allow for more frequent updates and better convergence.

The number of epochs required to achieve optimal prediction accuracy also showed variability, indicating the need for tailored training durations based on dataset complexity. BioBERT needed 5 epochs for the Revised JNLPBA dataset but 10 epochs for the BC5CDR dataset, suggesting that more training iterations are essential for specific datasets to fully capture their complexity and variability. Different datasets may demand varied training durations to achieve optimal performance.

The dropout rate, a critical parameter to prevent overfitting, exhibited significant variation across models and datasets. BioBERT, BigBird, and RoBERTa demonstrated better prediction accuracy with a lower dropout rate of 0.1 on the BC5CDR dataset, whereas a higher dropout rate of 0.5 was optimal for the Revised JNLPBA dataset. This variation underscores the differing overfitting risks and regularization needs across datasets. Higher dropout rates can prevent overfitting in more straightforward datasets but may hinder performance in more complex datasets requiring nuanced learning.

These findings emphasize the critical role of hyperparameter tuning in fine-tuning BERT-based models. While the ADAM optimizer with a learning rate of 0.0001 proved adequate, batch size, epochs, and dropout rate required careful adjustment to the specific characteristics of each dataset and model architecture. This variability highlights the necessity of a tailored approach in hyperparameter optimization to achieve optimal prediction accuracy. The consistent performance of deep learning models based on BERT architecture underscores their potential for significantly improving medical NER tasks. However, achieving optimal results across diverse medical contexts will require continuous refinement and adaptation of these models to the unique nuances of each dataset.

### Impact of Hyperparameters and Optimizers on the Fine-Tuning of the Gemma Model

The fine-tuning of the Gemma model using the LoRA technique led to significant improvements in its performance metrics across various medical NER tasks, as shown in Table 4. The consistent increases in AVG_MICRO metrics across 3 datasets demonstrate the effectiveness of LoRA in enhancing model accuracy. Although the AVG_MACRO metric declined in one dataset, it still improved in the other two, reaffirming LoRA's overall positive impact on model performance.

LoRA involves freezing the pretrained model weights and introducing trainable low-rank decomposition matrices in each transformer layer, significantly reducing the number of trainable parameters and GPU memory requirements. By optimizing these low-rank matrices, LoRA adapts the model to specific tasks without the computational burden of retraining the entire model. This technique focuses on adjusting the weights of hyperparameters rather than changing their values, allowing for more precise and efficient fine-tuning. The learning rate, managed using a cosine learning rate scheduler with a warm-up ratio of 0.1, ensures stable and efficient convergence. The consistent use of a learning rate of 0.0001 across various datasets demonstrated its effectiveness in balancing convergence speed and accuracy.

Hyperparameters such as batch size, epochs, and dropout rate were critical in determining model performance. Although their values remained constant during the LoRA fine-tuning process, the adjustments made by LoRA allowed the model to adapt effectively to the specific characteristics of each dataset. For instance, the Revised JNLPBA dataset benefited from a larger batch size and fewer epochs. In comparison, the BC5CDR dataset required a smaller batch size and more epochs to capture its complexities adequately. This variability underscores the importance of dataset-specific configurations in hyperparameter tuning to maximize model performance.

The dropout rate, crucial for preventing overfitting, also showed a significant impact depending on the dataset’s structure. For example, the Revised JNLPBA dataset, with its more straightforward structure, benefited from its original higher dropout rate, providing more robust regularization. Conversely, the BC5CDR and AnatEM datasets, with their more complex structures, yielded a better performance with their original lower dropout rates, allowing the model to retain more information during training. This finding highlights the necessity of understanding the specific regularization needs of each dataset to optimize model performance effectively.

The consistent improvement in AVG_MICRO and AVG_MACRO metrics demonstrates the enhanced generalization capability of the Gemma model after fine-tuning. These improvements are particularly significant for medical NER tasks, where precise entity recognition is critical for extracting meaningful information from complex texts. The results suggest that LoRA enables Gemma to capture nuanced patterns within the datasets more effectively, leading to better prediction accuracy and balanced performance across different entity types. Furthermore, the fine-tuning of the Gemma model using the LoRA technique highlights the critical role of weight adjustment in hyperparameter tuning and advanced optimization strategies in improving model performance. The significant improvements observed emphasize the potential of LoRA in efficiently adapting large-scale models to specialized tasks, making them more practical for deployment in various medical contexts.

### Evaluation of Resource Use Across NER Models

Gemma consistently required the most extended training times across all datasets, peaking at 63.34 minutes in the Revised JNLPBA dataset. This extended training duration reflects Gemma’s complex architecture, including numerous layers and parameters. The model’s intricate design requires significant processing power and time to adjust its parameters accurately through extensive training iterations.

Regarding CPU use, DeBERTa recorded the highest use in the Revised JNLPBA and AnatEM datasets, with use rates of 40.24% and 38.35%, respectively. DeBERTa’s high CPU use indicates its sophisticated architecture, which includes enhanced attention mechanisms and deeper layers compared to other models. These features demand substantial computational resources, leading to increased CPU consumption. The model’s reliance on complex attention mechanisms to capture fine-grained dependencies within the text likely contributes to its higher computational intensity, impacting its efficiency in resource-constrained environments.

In the BC5CDR dataset, CRF exhibited the highest CPU use, reaching 37.26%. This high CPU use can be attributed to the intensive computations required for feature extraction and sequence labeling in a dataset with relatively more straightforward entity types. While less complex than the deep learning models, the CRF model still requires significant CPU resources for processing and optimizing the conditional dependencies between the labels, especially when the dataset has clear, well-defined entity types that increase the computational workload.

In contrast, Gemma demonstrated minimal CPU requirements, maintaining use rates of approximately 10% across all datasets. This low CPU use suggests that Gemma offloads most of its computational workload to the GPU, leveraging its parallel processing capabilities to handle the model’s complex computations more efficiently. Gemma’s architecture is designed to exploit GPU parallelism, which allows for faster processing of large batches of data, thereby reducing the strain on the CPU. Thus, Gemma had the highest GPU use, consistently at approximately 61% across all datasets. This high GPU use underscores Gemma’s dependency on GPU resources to manage its computationally intensive tasks. The model’s architecture, characterized by numerous layers and a high parameter count, requires substantial GPU resources for the parallel processing needed during training and inference. The reliance on GPUs is due to their ability to handle multiple operations simultaneously, which is essential for deep learning models with large-scale parameters and complex computations.

BioBERT demonstrated the lowest GPU use among the BERT-based models, consuming 42.96% and 41.47% on the Revised JNLPBA and AnatEM datasets, respectively, and only 23.45% on the BC5CDR dataset. BioBERT’s lower GPU consumption can be attributed to its optimized architecture and efficient memory management, allowing it to balance computational demands with performance effectively. BioBERT’s design leverages GPU resources efficiently, ensuring that it can achieve high accuracy without excessively taxing computational resources.

Overall, Gemma’s significant resource requirements, evidenced by the fact that it had the highest GPU use and longest training times, highlight that its enhanced accuracy comes at a substantial cost in operational resources. The model’s advanced architecture necessitates extensive computational power to manage the large-scale parallel processing required for its numerous parameters and layers. This makes Gemma suitable for environments in which accuracy and abundant resources are paramount. In contrast, BioBERT’s combination of high prediction accuracy and lower resource consumption underscores its efficiency and suitability for environments with strict resource constraints. BioBERT’s ability to balance performance with resource use makes it versatile for various medical NER applications. These findings emphasize the importance of selecting NER models based on the deployment environment’s specific resource availability and operational constraints. Future research should focus on optimizing the computational efficiency of NER models without compromising their performance, ensuring that they can be effectively deployed across various medical contexts.

### Variability in Prediction Accuracy Across Entity Types in NER Models

Gemma demonstrated a superior performance in the entity categories of “chemical,” “disease,” “pathological formation,” and “immaterial anatomical entity,” likely due to its advanced design in feature extraction and semantic representation. Gemma uses optimized embedding techniques and attention mechanisms specifically tailored to manage the complexities inherent in these categories. For example, Gemma may use molecular property–based embedding representations in chemical entity recognition, enabling it to capture critical features of chemical substances. Gemma integrates rich medical ontologies and knowledge graphs in the context of diseases and pathological formations, enhancing its understanding of medical terminology and complex pathological descriptions. In addition, Gemma’s attention mechanisms focus on critical segments of the text, improving performance in recognizing immaterial anatomical entities.

BioBERT’s outstanding performance in the “organism substance” entity category can be attributed to its extensive pretraining in medical literature, particularly that covering organism substances. On the basis of the BERT bidirectional transformer architecture, BioBERT captures long-range dependencies in complex medical texts. The intricate language and contextual information in organism substance literature are effectively parsed by BioBERT’s architecture, leading to superior performance in this category. However, BioBERT’s performance in the “chemical” category was not as strong as Gemma’s, possibly because its pretraining data focus more on organism-related content than on chemical substances’ specific features.

DeBERTa’s superior performance in the “macromolecule” and “anatomical structure” entity categories was due to its innovative attention mechanisms and position encoding methods. Macromolecules and anatomical structures often exhibit high complexity and a hierarchical nature. DeBERTa’s disentangled attention mechanism allows for a fine-grained capture of internal relationships and hierarchical information within these complex structures. Its enhanced position encoding method effectively represents these complex medical entities, leading to an outstanding performance in these categories.

BigBird’s excellent performance in the “cell” entity category reflects its ability to handle long texts. Texts in cell biology are typically very detailed and information dense. BigBird’s architecture allows for more oversized context windows, enabling effective processing and analysis of these extensive texts. BigBird maintains efficiency and accuracy through its sparse attention mechanism, resulting in significant advantages in precisely classifying cell types.

This analysis reveals the necessity for a refined approach to improving medical NER models, emphasizing that, while extensive dataset training can improve the overall accuracy, architectural adjustments and targeted training are essential to mitigate disparities in model performance across diverse entity types. Therefore, enhancing the accuracy and robustness of NER models involves increasing the variety and volume of training data and optimizing model architectures to address the specific challenges posed by less represented or more intricate entity types.

### Influence of Macrofactors on Prediction Accuracy in Medical NER

In analyzing how macrofactor metrics influence prediction accuracy across various medical entity types within 7 NER models, we observed significant nuances that reflect the complexity of modeling in medical NER tasks. Notably, entities characterized by higher values in *sLen*, *eLen*, *eNum*, and *eDen* generally yielded better prediction accuracy. This correlation suggests that entities with more extensive and detailed textual representations tend to be predicted more accurately, highlighting the models’ capacity to handle intricate data structures effectively. However, a notable exception arises with *elCon*, which inversely correlated with these metrics, indicating a potential trade-off between detailed data processing and consistent label accuracy.

This phenomenon is further complicated by the varying impact of the *tEWC* on prediction accuracy across different datasets. For example, in the Revised JNLPBA dataset, lower *tEWC* values were associated with higher accuracies, suggesting that models perform better with more concise entity representations. In contrast, other datasets showed that higher *tEWC* values, indicative of richer contextual data, enhanced model performance. This inconsistency underscores the complex influence of data characteristics on model effectiveness and suggests that the optimal balance of data quantity and quality varies significantly across datasets. Therefore, tailored model training and data preparation strategies are essential to optimize prediction accuracy according to the unique characteristics of each dataset.

These observations necessitate a strategic approach to model training and data preparation that considers the unique demands of each dataset. While handling more extensive datasets can lead to better entity recognition in some contexts, balancing this with the need for precision and consistency in entity labeling is crucial. As NER technologies evolve, it becomes imperative to refine model architectures and training methodologies to ensure that models can manage the dual challenges of complexity and volume without sacrificing accuracy.

### Refining Macrofactor Sensitivity to Improve NER Model Precision

Using the MFE algorithm for hierarchical macrofactor screening, *eNum* or *eLen* in each entity phrase had the most significant impact on the prediction accuracy of NER models. Unlike broader textual metrics such as *sLen* that provide general context, *eNum* directly measures the complexity of entities, whereas *eLen* captures the length of entity phrases. These factors significantly influence how models process and interpret dense information. A higher *eNum* generally indicates semantically rich entities that are potentially more challenging to analyze. At the same time, a higher *eLen* suggests that entity phrases contain more detailed and extended descriptions requiring careful parsing.

The consistent selection of *eNum* or *eLen* in the final layer of the screening process across various datasets underscores their pivotal role in enhancing the precision of entity recognition. The impact of *eNum* on model accuracy suggests that entities with a higher density of words require sophisticated model capabilities to discern and categorize detailed information accurately. Similarly, entities with longer phrases (*eLen*) present a challenge as they involve more complex syntactic and semantic structures that must be interpreted correctly.

Therefore, refining models’ ability to analyze entities with higher *eNum* or longer *eLen* values could be a strategic approach to advancing NER technologies, particularly in domains such as medicine, where precise and reliable entity recognition is crucial. Enhancing how models manage and use the detailed information encapsulated in *eNum* and *eLen* will improve the robustness and effectiveness of medical NER models, ensuring that they meet the complex demands of varied and extensive datasets.

### Conclusions

Medical NER is a crucial component of medical informatics, essential for identifying and categorizing named entities within unstructured medical text data. A proficient NER model significantly enhances various downstream applications, such as medical text classification, question answering, and information retrieval. Developing a high-performance NER model requires a meticulous approach that includes selecting relevant macrofactors, designing the model’s architecture, and curating specialized training data tailored to medical contexts.

Our evaluation method for NER models extends beyond general metrics such as accuracy, recall, and *F*_1_-score by incorporating an extensive analysis of macrofactors relevant to medical entities. This comprehensive approach enables a multidimensional evaluation of the models, providing insights into how different entity types, attributes, and contextual factors influence performance. For example, our findings indicate that, while “disease” frequently occurs in medical texts and requires high accuracy, entities such as “Immaterial_anatomical_entity” may not require the same precision. This discrepancy highlights the need for targeted optimization strategies for different entity types, which is crucial for advancing medical NER models.

In addition, our study explored the characteristics of entities to improve the creation of high-quality medical NER datasets and documents. This focus enhances the NER models’ ability to identify entities accurately and addresses the specific needs of medical texts. While our analysis extensively covered macrofactors and their impact, it did not delve into misclassifications of entity labels or the fine-grained interactions between entity words. These areas could further refine our understanding of model accuracy. Moreover, examining hardware performance illuminates these models’ internal efficiency and resource use, which is crucial for their deployment in real-world scenarios.

In conclusion, evaluating medical NER models is essential for developing effective and precise NLP applications in health care. It gives medical researchers the insights to select and refine NER models suited to various medical scenarios, ultimately improving these systems’ accuracy, robustness, and reliability. This foundational work sets the stage for future research that could explore the intricate relationships within NER systems, further enhancing the capabilities of medical informatics.

## Abbreviations

ADAM: Adaptive Moment Estimation
AnatEM: Anatomical Entity Mention
AVG_MACRO: macroaverage
AVG_MICRO: microaverage
BERT: Bidirectional Encoder Representations From Transformers
BigBird: Big Transformer Models for Efficient Long-Sequence Attention
CPU: central processing unit
CRF: conditional random field
DeBERTa: Decoding-enhanced Bidirectional Encoder Representations From Transformers with Disentangled Attention
eDen: entity density
elCon: entity label consistency
eLen: entity phrase length
eNum: number of entity words in each entity phrase
GPU: graphics processing unit
HMM: hidden Markov model
JNLPBA: Joint Workshop on Natural Language Processing in Biomedicine and its Applications
LLM: large language model
LoRA: low-rank adaptation
MFE: multilevel factor elimination
NER: named entity recognition
NLP: natural language processing
RoBERTa: Robustly Optimized BERT Pretraining Approach
sLen: sentence length
tEWC: total entity word count in each entity type
